# Telomerase reverse transcriptase promotes angiogenesis in neonatal rats after hypoxic-ischemic brain damage

**DOI:** 10.7717/peerj.14220

**Published:** 2022-10-21

**Authors:** Jiao Li, Yi Feng, Jing Zhao, Zhi Fang, Haiting Liu

**Affiliations:** 1 Department of Pediatrics, West China Second University Hospital, Sichuan University, Chengdu, China; 2 Department of Cardiovascular Surgery, West China Hospital, Sichuan University, Chengdu, China

**Keywords:** Angiogenesis, Blood-brain barrier, Hypoxic ischemic brain damage, Neonate, Telomerase reverse transcriptase

## Abstract

**Background:**

Angiogenesis is an endogenous repair mechanism following hypoxic-ischemic brain damage (HIBD). Interestingly, recent studies have shown that angiogenesis can be regulated by telomerase reverse transcriptase (TERT), a critical component of telomerase. As telomerase reverse transcriptase can promote angiogenesis after stroke, we hypothesized that it could also promote angiogenesis after HIBD. To test this hypothesis, we developed *in vivo* and *in vitro* HIBD models in neonatal rats.

**Methods:**

TERT was overexpressed by lentivirus and adenovirus infection, and levels were measured using quantitative real-time polymerase chain reaction. We used a cell counting kit to quantify the proliferation rate of brain microvascular endothelial cells (BMECs), and immunofluorescence staining to measure CD34 expression levels. A microvessel formation assay was used to evaluate angiogenesis. Blood-brain barrier (BBB) integrity was assessed using immunohistochemical staining for ZO-1 and Evans Blue staining. Lastly, the expression level of Notch-1 was measured by western blotting.

**Results:**

Overexpression of *TERT* promoted the proliferation of BMECs after hypoxic-ischemic damage *in vitro*. *TERT* overexpression increased the formation of microvessels in the neonatal brain after HIBD both *in vivo* and *in vitro*. Overexpression of *TERT* improved BBB integrity in the brains of neonatal rats after HIBD. In addition, the expression level of Notch-1 was increased in BMECs following oxygen glucose deprivation, and overexpression of TERT further increased Notch-1 expression levels in BMECs following oxygen glucose deprivation.

**Discussion:**

Our results reveal that telomerase reverse transcriptase promotes angiogenesis and maintains the integrity of the blood-brain barrier after neonatal hypoxic-ischemic brain damage. Furthermore, the Notch-1 signaling pathway appears to contribute to the angiogenic function of telomerase reverse transcriptase. This protective effect of telomerase reverse transcriptase opens new horizons for future investigations aimed at uncovering the full potential of telomerase reverse transcriptase as a promising new target for the treatment of hypoxic-ischemic encephalopathy.

## Introduction

Hypoxic-ischemic encephalopathy (HIE) is one of the major causes of death and chronic nervous system injury in neonates. With no effective treatment, the only care available to neonates with HIE is symptomatic support. Recently, hypothermia has become the main treatment option for HIE as it has been proven to be neuroprotective. However, hypothermia has limited effects in improving the prognosis of HIE, and new therapeutic strategies are required (Douglas-Escobar & Weiss, 2015).

Angiogenesis, which refers to vascular remodeling and neovascularization, improves the oxygen and blood supply to injured tissues, and thus supports the repair of injured cells. Angiogenesis is one of the repair mechanisms after hypoxic-ischemic brain injury (Trollmann et al., 2018; Huang et al., 2004) and is involved in long-term recovery (Fernández-López et al., 2013). In addition, angiogenesis also supports the maintenance of the blood–brain barrier (BBB) integrity, which regulates cerebral homeostasis (Fernández-López et al., 2013).

Telomerase reverse transcriptase (TERT) is the catalytic subunit of telomerase that controls telomerase activity. As an important component of the telomerase complex, the major role of TERT is to maintain the length of telomere and thus maintain the genome stability (Blackburn, 2005; Wyatt, West & Beattie, 2010). However, previous studies have demonstrated that TERT has many other non-canonical functions, independent of its telomerase role, including anti-apoptosis, excitotoxicity defense, promoting angiogenesis and neurogenesis, regulating gene expression and signaling pathways related with cell proliferation and death (Chung et al., 2005; Li et al., 2011). A telomerase-independent role of TERT in regulating angiogenesis has been described in nervous system tumors and ischemic injuries (George, Banik & Ray, 2009; Zaccagnini et al., 2005). Previous studies have already reported that TERT overexpression promotes the proliferation and microvessel formation of normal endothelial cells (Liu et al., 2016; Maishi et al., 2019). In a mouse model of middle cerebral artery occlusion, inhibition of TERT aggravated the injury to the microvessels and BBB, indicating that TERT is involved in the regulation of angiogenesis and BBB function after ischemic brain injury (Zhang et al., 2010).

Based on these studies, we hypothesized that TERT might also be involved in promoting angiogenesis after neonatal hypoxic-ischemic brain damage (HIBD). In this study, we developed both *in vivo* and *in vitro* models of neonatal HIBD and overexpressed *TERT* using lentivirus and adenovirus infection to test this hypothesis.

We also investigated the possible signaling pathway for TERT to regulate angiogenesis during HIBD. The Notch signaling pathway is a key pathway in the normal process of vascular development, which is also involved in the neo-vascularization after ischemic injury (Al Haj Zen & Madeddu, 2009). In this study, we determined the expression of Notch-1 in brain microvascular endothelial cells after hypoxic ischemic injury.

## Materials & Methods

### Experimental animals

The number of experimental animals for each group was calculated using power analysis sample size (PASS) software (version 15; NCSS, LLC., Kaysville, Utah, USA), based on a power of 90% and a *p*-value < 0.05. With the approval of the Sichuan University Committee of Animal Research, neonatal Sprague Dawley (SD) rats (30 rats, 15 males and 15 females, 5 days old, weight of 15–20 g) were provided by the Animal Center of Sichuan Province. Animal experiments were performed in compliance with the protocols approved by the Animal Ethics Committee of West China Second University Hospital, Sichuan University (approval number, 2021034). All animal experiments in this study complied with the animal research: reporting of *in vivo* experiments (ARRIVE) guidelines (Kilkenny et al., 2010) and were carried out in accordance with the recent guidelines (Percie du Sert et al., 2020). Furthermore, the work in this study followed the U.K. Animals (Scientific Procedures) Act, 1986 and the EU Directive 2010/63/EU for animal experiments.

Before experiments, all neonatal rats were housed at room temperature (22  ± 2 °C) with their mother rat and were allowed free access to food and water. Their vital signs were monitored, and pain was evaluated based on appearance, voice, and dietary changes. Buprenorphine was administered as required, during experiments, to reduce pain. After all the experiments (at 72 h or 2 weeks after hypoxic-ischemic treatment depending on the experiment), animals were euthanized *via* cervical dislocation following anesthesia.

Overall, 27 SD rats were included in the experiment, as three neonatal rats died after 2 days of adaptive feeding. The 27 SD rats were randomly assigned to the three experimental groups (*n* = 9 each): sham, HIBD + vehicle, and HIBD + TERT.

### Brain microvascular endothelial cells (BMECs) cultured *in vitro*

Brain microvascular endothelial cells (BMECs) from Procell (Procell Life Science & Technology Co., Ltd., Wuhan, China) were cultured in Dulbecco’s Modified Eagle Medium (DMEM; Hyclone Laboratories, Inc., South Logan, UT, USA) supplemented with 10% fetal bovine serum (Thermo Fisher Scientific, Waltham, MA, USA) in a humidified atmosphere at 37 °C (95% air and 5% CO_2_). Cells were passaged for 2–3 days.

The *in vitro* experimental groups included the following: BMECs infected with empty vector lentivirus and exposed to oxygen glucose deprivation (OGD + empty) or cultured in normoxia conditions (control). BMECs with lentivirus-mediated overexpression of TERT were exposed to OGD (OGD + TERT). All the cultured BMECs were randomly assigned to the three experimental groups by a researcher who was blinded to the study.

### Construction of vectors and lentiviral infection of BMECs

The *TERT* gene was synthesized according to the CDS sequence (https://www.ncbi.nlm.nih.gov/nuccore/NM_053423.1) and inserted into vector. The lenti-vector system was 3rd: pCDH–CMV-MCS-EF1-copGFP (Wuhan GeneCreate Biological Engineering Co., Ltd.). The empty vector was used as the control plasmid. After transformation, validation, and amplification, two types of plasmids were packaged with human embryonic kidney 293T cells. The final supernatant (containing either *TERT* overexpression vector or empty vector) was collected after filtration.

Cultured BMECs were digested with 0.125% trypsin and seeded onto a 24-well plate. The BMECs were infected with modified lentivirus at a multiplicity of infection of 20. After infection for 4 h, OGD treatment was performed.

### Oxygen glucose deprivation treatment

A mixture of 95% N_2_ and 5% CO_2_ was used to replace the O_2_ in glucose-free DMEM. After being washed with glucose-free DMEM, the BMECs were placed into Billups-Rothenberg incubation chambers with continuous mixed gas (95% N_2_ and 5% CO_2_) for 4 min at a flow velocity of 10 L/min. The cells were then incubated in a hypoxic airtight chamber with 95% N_2_ and 5% CO_2_ at 37 °C. After 4 h of OGD treatment, the cells were returned to normal conditions with glucose and oxygen for 48 h.

### Infection with adenovirus and hypoxia-ischemia treatment *in vivo*

Two types of adenovirus vectors were used in this study: the adenovirus-mediated overexpression of TERT vector and the empty adenovirus vector. Both were provided by ZHBY (ZHBY Biotech Co., Ltd., Nanchang, China) and identified by quantitative real-time polymerase chain reaction (PCR) . Nine rats in each group were randomly selected by a researcher blinded to the study procedure. In each group of rats, 2 µL of *TERT*-overexpressing adenovirus, empty adenovirus vector, or saline was injected into the lateral cerebral ventricle.

After 48 h of infection, the rats, injected with *TERT*-overexpressing adenovirus and empty adenovirus vectors, underwent hypoxic-ischemic treatment, while those with saline injection served as a control (sham). The HIBD model was established *in vivo* as follows:

Neonatal rats were anesthetized by inhaled isoflurane, which is safe and quickly metabolized. Initial anesthesia was induced *via* inhalation of 4% isoflurane (RWD Life Science Co., LTD, Shenzhen, China), and then maintained *via* continuous inhalation of 2% isoflurane for surgery. The rats were fixed on a surgical plate during the surgery. The skin on the neck was incised after disinfection, and the right carotid artery was identified and ligated. After surgery, the rats were returned to their mothers for 2 h. The rats were then placed in chambers at 37 °C containing 8% O_2_ for 2.5 h. After hypoxic-ischemic treatment, rats were again removed for normal feeding. Sham control rats underwent the same surgical procedure without hypoxic-ischemic treatment.

Based on the treatments above, all rats were randomly divided into three groups: sham (rats injected with saline into the lateral cerebral ventricle with no hypoxic-ischemic treatment), HIBD + vehicle (rats injected with empty adenovirus vector into the lateral cerebral ventricle and subjected to hypoxic-ischemic treatment), and HIBD + TERT group (rats injected with TERT-overexpressing adenovirus into the lateral cerebral ventricle and subjected to hypoxic-ischemic treatment). The researcher responsible for the experimental grouping did not participate in the subsequent experiments, and other researchers who were unaware of the animal’s group performed the subsequent procedures and statistical analysis.

### Quantitative real-time PCR

Total RNA was extracted from the BMECs using an Ultrapure RNA Kit (DNaseI, CWBIO, Jiangsu, China) to detect *TERT* expression levels. Primers for *TERT* were designed (General Biol, Anhui, China) (primer sequences: TERT-F TCTTGTCAGTCTTGCGGTTGA; TERT-R CAAAGGGAAGCCGAATCACAC), and the HiFiScript cDNA synthesis kit (CWBIO, Jiangsu, China) was used to synthesize the first-strand cDNA. Amplification was performed at 95 °C for 10 min, followed by 40 cycles at 95 °C for 10 s and at 60 °C for 30 s with UltraSYBR Mixture (CWBIO, Jiangsu, China).

Information about the position of the amplification was: NM_053423.1 Rattus norvegicus telomerase reverse transcriptase (Tert), mRNA, 2900-3043, amplification length 144bp. The final data were analyzed using the 2- Δ ΔCt method which was normalized to a housekeeping gene.

### Cell counting and kit-8 staining

BMECs were seeded into 96-well plates at a density of 1–5 × 10^4^ cells/mL and cultured at 37 °C overnight. The culture medium of the BMECs was replaced with the mixture of a cell counting kit (CCK8, Dojindo, Kumamoto, Japan) and serum-free DMEM for 1 h at 37 °C. The absorbance value (A) was measured at 450 nm using a microplate reader (Presong, Beijing, China). The proliferation rate was calculated using the following equation:

Proliferation rate of BMECs = A in the experimental group/A value in the control group × 100%.

### Western blot

RIPA lysis buffer (Thermo Fisher Scientific, Waltham, MA, USA) was added to the BMECs in each group. Protein supernatants were collected after centrifugation at 12,000 rpm and 4 °C for 15 min. The protein was denatured by boiling for 5 min. Protein concentration was determined using the bicinchoninic acid Protein Assay Kit (CWBIO, Jiangsu, China). Equal amounts of the remaining protein samples then underwent 10% sodium dodecyl sulfate/polyacrylamide gel electrophoresis before being electrotransferred onto a polyvinylidene fluoride membrane (Roche, Basel, Switzerland). After blocking with 5% blocking buffer (Solarbio, Beijing, China), membranes were incubated with primary antibodies against Notch-1 (1:1000, Abways Technology, Inc., Shanghai, China) overnight. After washes, membranes were incubated with horseradish peroxidase-conjugated secondary antibody (1:200, Abcam Plc., Cambridge, U.K.), washed again, treated with an enhanced chemiluminescence kit (EMD Merck Millipore, Billerica, MA, USA), and exposed to the Chemi-DOC^TM^XR + system (Bio-Rad Laboratories, Hercules, CA, USA) for autoradiography. The *β*-actin protein (Bioss, Woburn, MA, USA), was used as an internal reference. The TERT to *β*-actin optical density ratio was calculated for statistical analysis. All experiments were repeated three times.

### Immunofluorescence staining

The expression of CD34 reflects the density of microvessels in the brain (Zhang et al., 2019) as CD34 is a marker of endothelial cells. Therefore, CD34 levels in the brain were measured by immunofluorescence staining to determine the formation of microvessels. After perfusion with 30 mL saline and gradual fixation with 10 mL 4% paraformaldehyde, the brains of the rats in all the groups were removed and fixed again with 4% paraformaldehyde for 48 h. The brains were then embedded in 4% agarose, then frozen and cut into frozen sections of 50 µm thickness. Then, 0.3% phosphate-buffered saline plus Triton X-100 (Solarbio, Beijing, China) was added for 10 min. After blocking with 5% fetal bovine serum, the sections were incubated with primary antibodies against CD34 (1:100, Abcam Plc., Cambridge, U.K.) at 4 °C, overnight. On the second day, after phosphate-buffered saline washes, brain sections were incubated with FITC 647-conjugated secondary antibodies (Invitrogen, Carlsbad, CA, USA) for 2 h at room temperature. The brain sections were then stained with 4′,6-diamidino-2-phenylindole (DAPI) and washed with phosphate-buffered saline. The final sections were produced by fixing with an anti-fluorescence quenching agent (Beyotime, Shanghai, China). A fluorescence microscope (Nikon Eclipse 80i; Nikon Corporation, Tokyo, Japan) was used to obtain images. A minimum of three slices (5 µm thick) per brain were captured using the 20x objective. The analysis was conducted at the same position in all brain sections in each group. Images were analyzed using Image-Pro Plus 6.0 (Media Cybernetics Inc., Rockville, MD, USA).

### Immunohistochemical assay

A tight junction protein of the BBB, ZO-1, participates in the maintenance of BBB integrity (Daneman & Prat, 2015). Thus, to evaluate the integrity of the BBB after HIBD, immunohistochemical assays were performed to analyze the expression of ZO-1. Frozen brain sections were prepared in the same manner as for immunofluorescence staining. The sections were treated with 3% H_2_O_2_for 15 min. After being washed with phosphate-buffered saline three times, the sections were blocked with 5% fetal bovine serum for 1 h at 37 °C. The brain sections were incubated with primary antibodies against the ZO-1 rabbit polyclonal antibody (1:500, Proteintech, Rosemont, IL, USA) overnight at 4 °C. On the second day, the brain sections were incubated with a horseradish peroxidase-conjugated secondary antibody (1:200, Boster Biological Technology, Wuhan, China) for 2 h. In the final step, 3,3-diaminobenzidine (KPL Inc., Gaithersburg, MD, USA) was used to visualize the immunoreactivity under a microscope (Olympus Corporation, Tokyo, Japan). Ten microscopic fields were randomly selected in each group to measure the integrated optical densities using Image-Pro Plus 6.0 (Media Cybernetics Inc., Rockville, MD, USA) software for analysis.

### Microvessel formation assay

Angiogenesis by the BMECs was investigated using a microvessel formation assay in vitro. Briefly, the BMECs were infected with empty oligonucleotides or *TERT* overexpression vectors. After 48 h, all BMECs in the three groups were digested with trypsin and then seeded at a density of 1 × 10^5^ cells/well into 24-well plates coated with 250 µL Matrigel Basement Membrane Matrix (BD Biosciences Pharmingen Inc., San Diego, CA, USA). After 5 h of culture, BMECs in the OGD + empty and OGD + TERT groups were exposed to OGD treatment. Twelve hours after reperfusion, samples from each group were imaged with an optical microscope (Olympus Corporation, Tokyo, Japan).

### Evaluation of blood–brain barrier permeability

Evans Blue (EB) was used to evaluate the permeability of the BBB. The rats were injected with 2% EB (two mL/kg) through the tail vein. After a 3 min waiting period, the rats were anesthetized and sacrificed. Brain tissues were weighed and soaked in formamide (one mL/100 mg brain tissue) in a water bath at 60 °C for 24 h. A spectrophotometer was used to measure the absorbance of the supernatant at 610 nm. The concentration of EB was analyzed using the standard curve.

**Figure 1:**
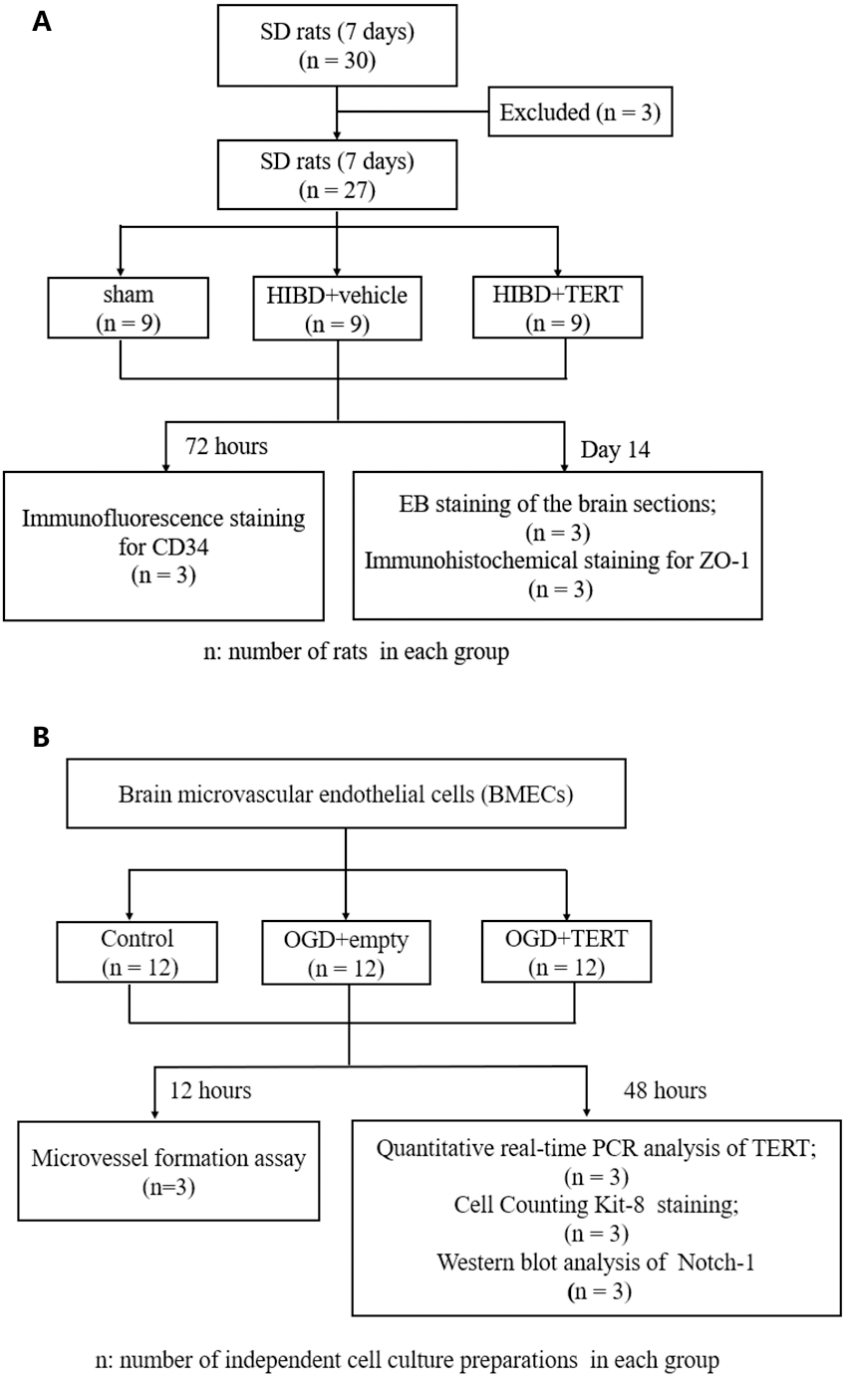
Flow chart of the experimental procedure. (A) The procedure of the *in vivo* experiment. (B) The procedure of the *in vitro* experiment. SD, Sprague-Dawley; HIBD, hypoxic-ischemic brain damage; TERT, telomerase reverse transcriptase; EB, Evans Blue; BMECs, brain microvascular endothelial cells; OGD, oxygen glucose deprivation.

Figure 1 shows the flow chart of the experimental procedure.

### Statistical analysis

SPSS 16.0 (IBM SPSS Statistics, USA) was used for statistical analysis. Data were expressed as the mean ± standard deviation. Analysis of variance with the LSD *post hoc* test was used to detect whether there were any significant differences among groups. Statistical significance was set at *P* < 0.05.

## Results

### *TERT* overexpression increases the proliferation rate of BMECs after OGD

**Figure 2:**
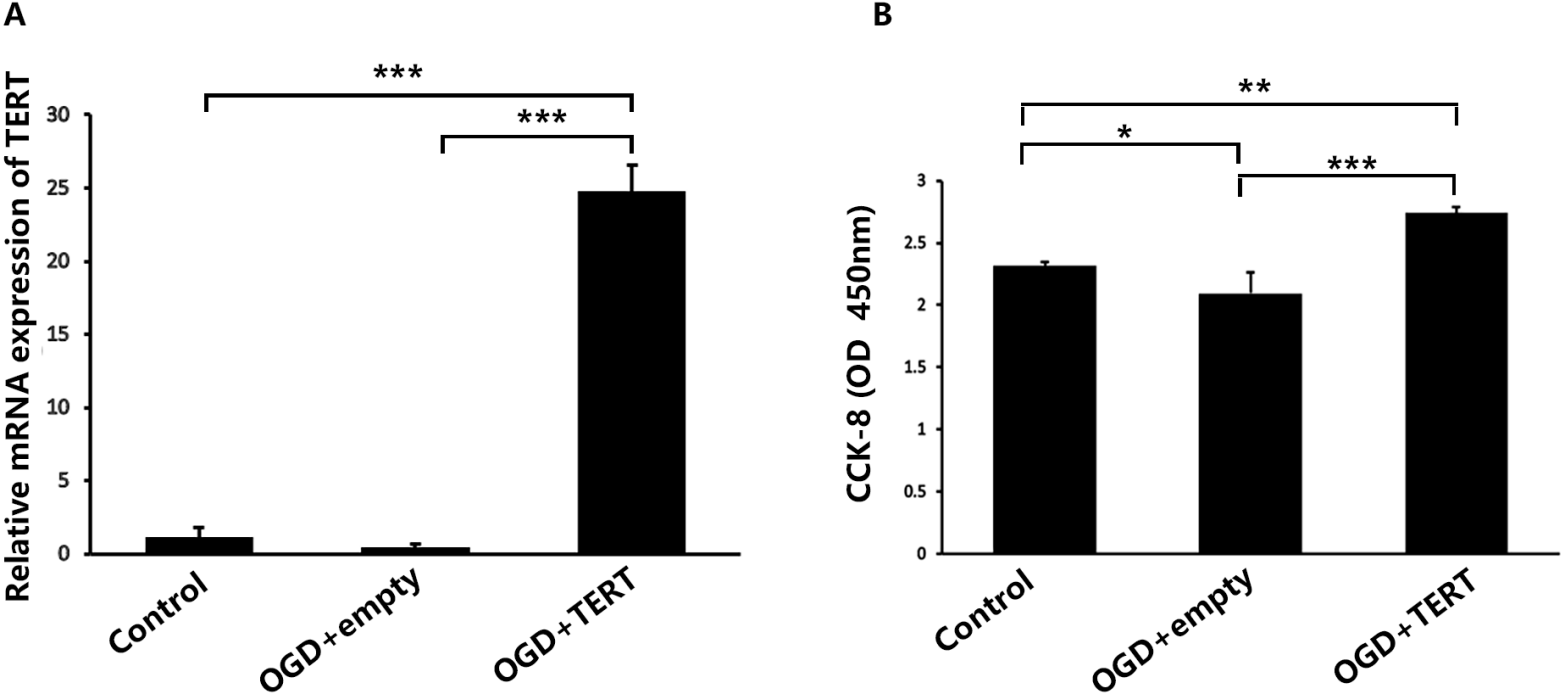
Telomerase reverse transcriptase expression and the proliferation rate of BMECs in the three study groups *in vitro*. (A) The mRNA level of *TERT* was detected with quantitative real-time polymerase chain reaction in the three BMECs groups. There was no significant difference between the control group and OGD + empty group. ***, *P* < 0.001 (*n* = 3 independent cell culture preparations in each group). (B) The optical density value (450 nm) determined with a cell counting assay in the three groups. *, *P* < 0.05; **, *P* < 0.01; ***, *P* < 0.001 (*n* = 3 independent cell culture preparations in each group). TERT, telomerase reverse transcriptase; BMECs, brain microvascular endothelial cells; OGD, oxygen-glucose deprivation.

Firstly, *in vitro TERT* overexpression was validated. Two days after OGD, cultured BMECs in three groups were collected for the following experiments. Figure 2A shows the relative mRNA expression level of *TERT* in the three groups. The *TERT* mRNA levels were significantly higher in the OGD + TERT group than in the OGD + empty group (*P* = 0.0000002, Fig. 2A), indicating that we had developed a valid *TERT* overexpression model in the BMECs after OGD. CCK8 assays were conducted to test the proliferation of BMECs after OGD. As shown in Fig. 2B, the proliferation rate of BMECs decreased after OGD (*P* = 0.039, control group *vs* OGD + empty group, Fig. 2B). However, the proliferation rate of BMECs in the OGD + TERT group was higher than that in the OGD + empty group (*P* = 0.0002, Fig. 2B).

### *TERT* overexpression increases the formation of microvessels in the brain after HIBD both *in vivo* and *in vitro*

**Figure 3:**
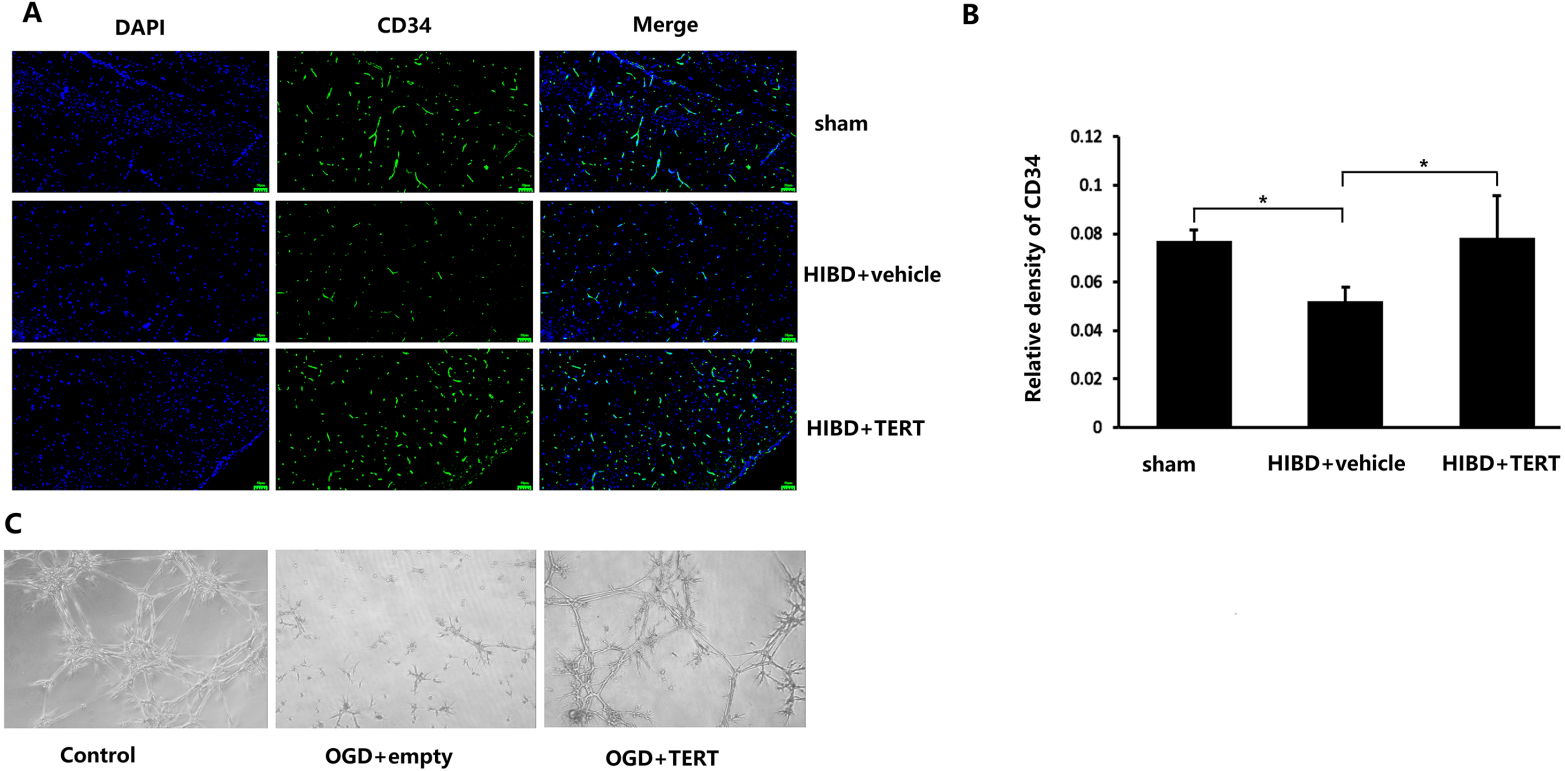
Telomerase reverse transcriptase promotes the formation of microvessels in a neonatal rat brain model after hypoxic-ischemic brain damage both *in vivo* and *in vitro*. (A) Immunofluorescence staining of CD34 (green) in the brains of the three groups *in vivo*. Nuclei were labeled with DAPI. Scale bar: 50 µm. (B) The immunoreactivity of CD34 was quantified. There was no significant difference between the sham group and HIBD + TERT group. *, *P* < 0.05 (*n* = 3 animals in each group). (C) *In vitro* comparison of microvessel formation assay in the three groups of brain microvascular endothelial cells. DAPI, 4′,6-diamidino-2-phenylindole; HIBD, hypoxic-ischemic brain damage; TERT, telomerase reverse transcriptase; OGD, oxygen-glucose deprivation.

To determine the microvessel density in the three groups, 72 h after hypoxic-ischemic treatment, the rats were sacrificed, and CD34 levels were measured. As shown in Figs. 3A and 3B, the microvessel density (CD34-positive cells) in the brain was significantly lower in the HIBD + vehicle than in the sham group (*P* = 0.032, Figs. 3A and 3B), while the microvessel density in HIBD + TERT group was significantly higher than in the HIBD + vehicle group (*P* = 0.026, Figs. 3A and 3B). With regard to the role of TERT in angiogenesis using the *in vitro* microvessel formation assay, fewer microvessels were observed in both the groups that were subjected to OGD than in the control group (Fig. 3C). However, more microvessels were found in the OGD + TERT group than in the OGD + empty group (Fig. 3C).

### TERT overexpression improves BBB integrity after HIBD

**Figure 4:**
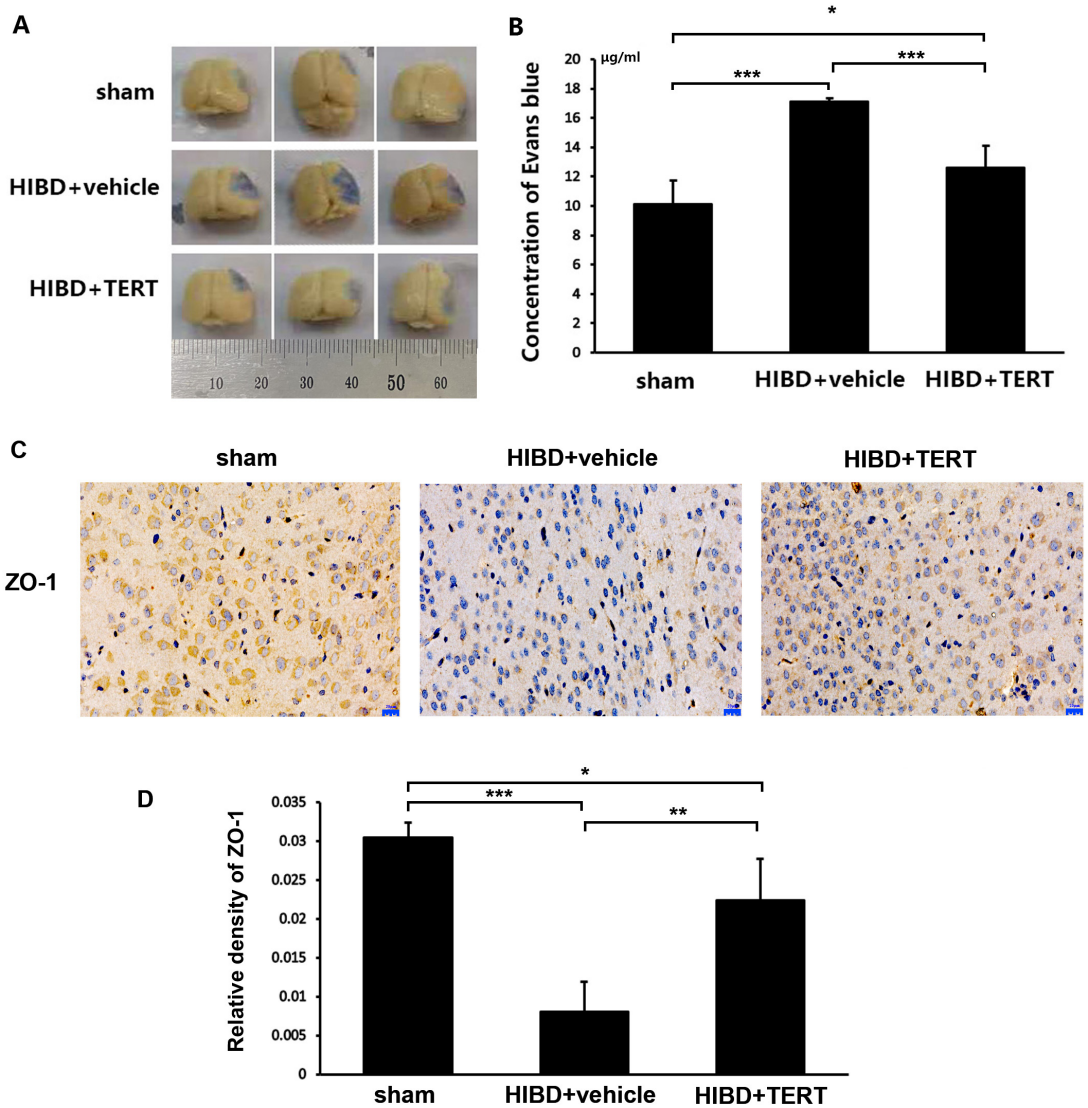
Telomerase reverse transcriptase promotes blood-brain barrier integrity after hypoxic-ischemic brain damage. (A) Evans Blue staining of the brain in the three study groups. (B) Quantitative analysis of Evans Blue concentration in the brain. *, *P* < 0.05; ***, *P* < 0.001 (*n* = 3 animals in each group). (C) Immunohistochemical staining of ZO-1 in the brains of the three study groups. Scale bar: 20 µm. (D) The immunoreactivity of ZO-1 in three groups. *, *P* < 0.05; **, *P* < 0.01; ***, *P* < 0.001 (*n* = 3 animals in each group). TERT, telomerase reverse transcriptase; HIBD, hypoxic-ischemic brain damage.

Two weeks after hypoxic-ischemic treatment, the rats were sacrificed for the measurements of BBB integrity and the expression level of ZO-1. Figures 4A and 4B show the EB staining of the brain sections in the three groups. The EB concentration was significantly higher in the HIBD + vehicle group than in the sham group (*P* = 0.0000007, Figs. 4A and 4B). Overexpression of *TERT* (HIBD + TERT) resulted in a significant reduction of EB concentration as compared to the HIBD + vehicle group (*P* = 0.00005, Figs. 4A and 4B). Furthermore, the expression of ZO-1 was much lower in both groups subjected to HIBD than in the sham group (*P* = 0.0005 sham group *vs* HIBD + vehicle group, *P* = 0.047 sham group *vs* HIBD + TERT group, Figs. 4C and 4D). However, the expression level of ZO-1 was significantly increased in the HIBD + TERT group as compared to the vehicle group (*P* = 0.005, Figs. 4C and 4D).

### TERT increases Notch-1 levels in BMECs following oxygen glucose deprivation

**Figure 5:**
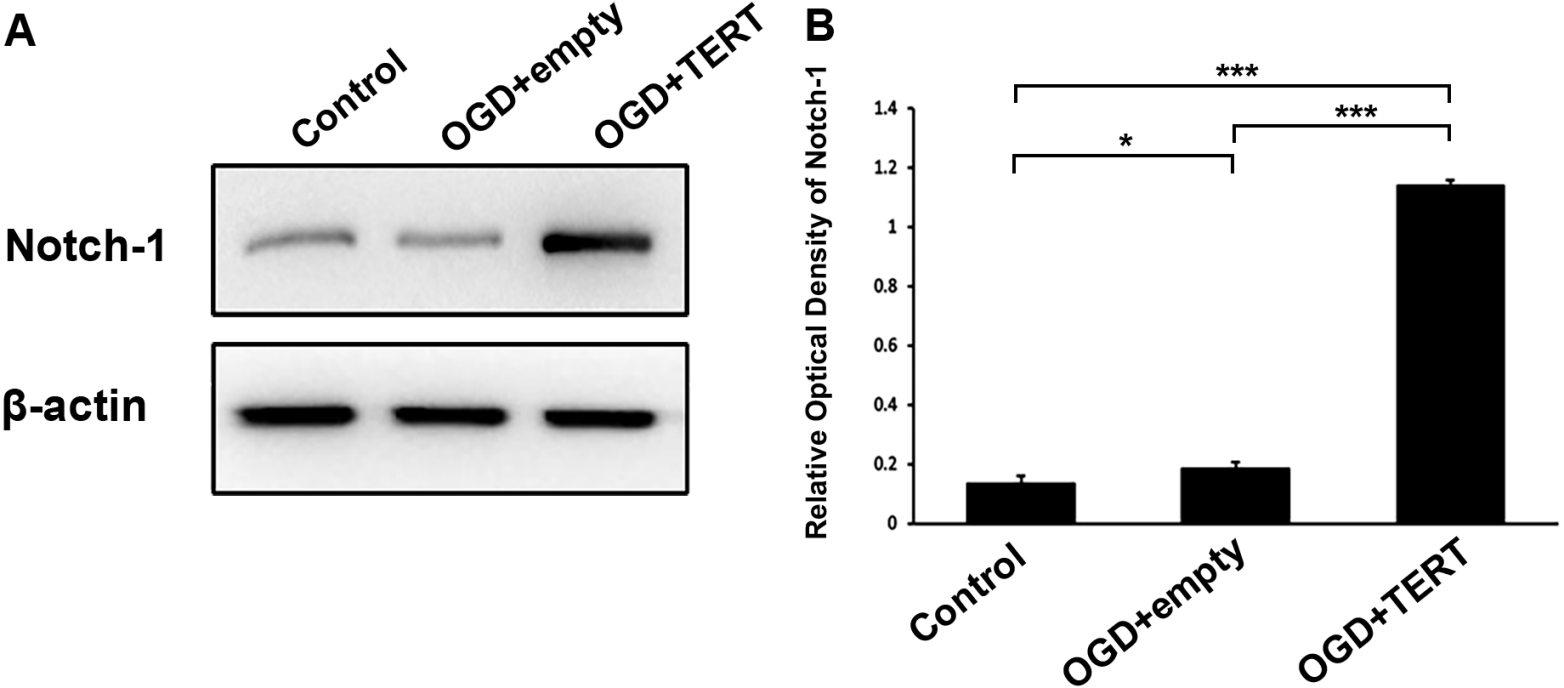
Telomerase reverse transcriptase enhances the expression of Notch-1 in BMECs after OGD. (A) Western blot detection of Notch-1 expression level in the three groups. (B) Quantification analysis of the Notch-1 expression using *β*-actin as the internal control. *, *P* < 0.05; ***, *P* < 0.001 (*n* = 3 independent cell culture preparations in each group). OGD, oxygen glucose deprivation; TERT, telomerase reverse transcriptase.

To investigate the mechanism underlying TERT-induced angiogenesis, we measured the levels of Notch-1 in BMECs at 48 h after OGD. As shown in Figures 5A and 5B, the expression of Notch-1 in the OGD + empty group was higher than in the control group (*P* = 0.021, Figs. 5A and 5B). Furthermore, Notch-1 expression in the OGD + TERT group was significantly higher than in the OGD + empty group (*P* = 0.0000000002, Figs. 5A and 5B). Thus, TERT enhanced the expression of Notch-1 in BMECs after OGD.

## Discussion

In this study, we found that overexpression of *TERT* promoted the proliferation of BMECs after hypoxic-ischemic damage *in vitro*. *TERT* overexpression increased the formation of microvessels in the neonatal brain after HIBD both *in vivo* and *in vitro*. Overexpression of *TERT* improved BBB integrity in the brains of neonatal rats after HIBD. In addition, the expression level of Notch-1 was increased in BMECs following oxygen glucose deprivation, and overexpression of TERT further increased Notch-1 expression levels in BMECs following oxygen glucose deprivation. We previously investigated the neuroprotective role of TERT in neonatal HIBD (Li et al., 2013; Zhao et al., 2012). However, little is known about the role of TERT in angiogenesis during neonatal HIBD. This study indicated that TERT can promote angiogenesis in neonatal rats after hypoxic-ischemic brain damage, which may open new horizons for future investigations aimed at uncovering the full potential of TERT as a promising new target for the treatment of HIE.

The functions of the nervous and vascular systems are closely linked in mammals. The vascular system plays a critical role in brain development because it provides blood supply and metabolic energy to the central nervous system. This kind of neurovascular crosstalk starts during embryonic development and continues throughout life (Ma & Zhang, 2015). After a brain injury, in addition to providing oxygen and nutrients to the injured area, angiogenesis is also believed to promote oligogenesis and myelination and, consequently, contribute to neural recovery (Wang et al., 2020). In a neonatal rat model of stroke, ischemia led to reduced endothelial cell proliferation and vessel degeneration, and the suppression of angiogenesis disrupted recovery (Fernández-López et al., 2013). Consequently, angiogenesis has become an interventional target in the treatment of HIE (Dixon et al., 2015). Indeed, improving angiogenesis can promote neural recovery from HIE in neonatal rats (Yan et al., 2016).

TERT has been widely studied in cellular senescence and tumor research. Angiogenesis is believed to be one of the telomerase-independent functions of TERT in tumors (Zou, Cong & Zhou, 2020). Inhibition of TERT effectively reduces angiogenesis in glioblastoma (George, Banik & Ray, 2009). In addition, TERT was shown to promote angiogenesis in an ischemic rat hind limb model (Zaccagnini et al., 2005). Transfection of TERT to normal endothelial cells increased their proliferation ability, kept their endothelial cell characteristics, and did not lead to any tumorigenic potential (Maishi et al., 2019). In normal cultured human umbilical vein endothelial cells (HUVECs), increased expression of TERT promoted the vascular tube formation, whereas inhibition the expression of TERT impaired the vascular tube formation (Liu et al., 2016). Their research also demonstrated that ectopic expression of TERT promoted the vascular formation and migration of HUVECs, which can finally promote angiogenesis *in vivo* (Liu et al., 2016). However, little is known about the angiogenesis-promoting role of TERT in a neonatal HIBD model. Since angiogenesis includes endothelial cell proliferation and microvessel formation (Su et al., 2019), we investigated the proliferation of endothelial cells *in vitro* and the formation of microvessels both *in vivo* and *in vitro*. Our data show that TERT can promote endothelial cell proliferation and microvessel formation in the neonatal brain after HIBD, thereby confirming our hypothesis. To our knowledge, this is the first study to demonstrate the angiogenic role of TERT in neonatal HIBD, which may open new avenues for the research of TERT in HIBD.

The BBB is a unique microvasculature in the central nervous system, which regulates the movement of molecules between the brain and blood and maintains central nervous system homeostasis. Various neurological diseases, including stroke and brain trauma, can cause BBB dysfunction and lead to neuronal injury (Daneman & Prat, 2015). BBB integrity is also affected during HIE. Hypoxic-ischemic damage can further increase BBB permeability, which aggravates brain edema and increases inflammation of the brain, which in turn exacerbates HIBD (Lee, Michael-Titus & Shah, 2017). In addition, BBB dysfunction in the ischemic brain can also suppress angiogenesis and neurogenesis during post-injury recovery (Fernández-López et al., 2013). These interactions, between the BBB and HIBD, thus made BBB a therapy target in HIE (Lee, Michael-Titus & Shah, 2017; Disdier & Stonestreet, 2020).

TERT has been reported to be involved in the modulation of BBB integrity during ischemic stroke (Zhang et al., 2010). However, little is known about the effect of TERT on the BBB during HIBD. In the present study, we investigated the impact of TERT on BBB function during neonatal HIBD. BBB permeability was measured by EB staining and immunohistochemical staining for ZO-1. The EB staining assay of the brain has been widely used to estimate BBB permeability (Li et al., 2020). ZO-1 is a tight junction protein in the brain that has been used to evaluate the integrity of the BBB in many previous studies (Erikson et al., 2020; Wang et al., 2016). The results of our study showed that TERT can reverse the BBB dysfunction induced by HIBD and that TERT can promote BBB integrity in the brains of neonatal rats after HIBD.

Our study demonstrated that TERT promotes angiogenesis in the brain and maintains BBB integrity after neonatal HIBD. However, the neuroprotective role of angiogenesis during HIBD remains controversial. Angiogenesis after HIE might lead to hyperperfusion injury, which is related to poor neurological outcomes (Kleuskens et al., 2021). It has been reported that the development of new microvessels may increase BBB permeability and aggravate brain edema (Adamczak & Hoehn, 2015). Although we did not measure brain edema in this study, BBB permeability decreased due to the overexpression of TERT, suggesting that TERT-induced angiogenesis is neuroprotective. However, further studies are needed to clarify the relationship between angiogenesis and BBB during HIBD.

Furthermore, we investigated the mechanism of how TERT promotes angiogenesis. The Notch signaling pathway is a highly conserved intracellular pathway that functions in the regulation of neural development and in the pathology of central nervous system diseases (Guo et al., 2021). Moreover, it is a key pathway in the regulation of angiogenesis after ischemic diseases (Al Haj Zen & Madeddu, 2009; Lou et al., 2012) that promotes endothelial cell migration, vessel structure formation, and vascular maturation (Lou et al., 2012). Thus, we determined the expression of Notch-1 *in vitro* and observed an increase in Notch-1 expression in BMECs after OGD. Overexpression of *TERT* caused a significant upregulation of Notch-1 levels in BMECs after OGD, which may further promote endothelial cell proliferation and microvessel formation. These data suggest that the Notch-1 signaling pathway may play a role in the angiogenic effect of TERT in neonatal HIBD. The connection between TERT and Notch-1 signaling pathway is still unclear. It is reported that Notch-1 can regulate TERT expression in tumors (Sawangarun et al., 2018). Giunco et al. (2015) reported that ectopic expression of TERT can increase the expression of Notch2 in transcription level, thus promote the Notch signaling pathway in lymphoblastoid cell lines in the research of Epstein–Barr virus-associated malignancies. However, further investigations are required to elucidate this mechanism, which may focus on the detection of the downstream targets of Notch signaling pathways and investigate whether inhibition of Notch-1 expression can reverse the angiogenesis-promoting effect of TERT during HIBD.

TERT expression can be widely detected in the developing brain inlcuding the embryonic period and early life after birth, but decreases soon after birth (Fu et al., 2000; Klapper, Shin & Mattson, 2001). In the adult brain, TERT is only expressed in the highly proliferative regions (Lee et al., 2010). Our studies have demonstrated that TERT plays a neuroprotective role in the neoantal brain after hypoxic ischemic brain damage (Li et al., 2022; Li et al., 2013), therefore how to upregulate the expression of TERT is critical. Researches including basic and clinical studies have been investigating the strategies to increase TERT expression to treat disorders. In addition to the method of transfering TERT gene, some antioxidant drugs may increase TERT activity (Madonna et al., 2011). It is reported that N-acetyl-cystein can increase TERT activity in endothelial cells (Voghel et al., 2008). Telomerase activtors including TA-65, an extract from Astragalous membranaceous and GRN510, a small molecule based on cycloastragenol have been proved to upregulate TERT expression effectively (Alshinnawy et al., 2021; Wan et al., 2021). Although whether these TERT activators can upregulate TERT expression in HIBD and thus exert a protective effect has not been confirmed, it gives us insight for subsequent clinical studies.

Our research had some limitations. In this study, we did not set an experimental group comprising normal BMECs and rats with *TERT* overexpression under normal conditions. This was mainly because the primary aim of this study was to investigate the role of TERT on angiogenesis during HIBD in neonatal rats, and previous studies have already reported that *TERT* overexpression drives the proliferation and microvessel formation of normal endothelial cells (Maishi et al., 2019; Liu et al., 2016). Another limitation of our research is lack of a scrambled control for TERT over-expression group, which will be supplemented in our future research. We have preliminarily explored the role of Notch-1 signaling pathway in the angiogenic effect of TERT in neonatal HIBD. However, interventions including overexpressing and inhibition of Notch-1 is needed to further validate the Notch-1 signaling pathway.

## Conclusions

Previous studies indicated that TERT promotes angiogenesis. This function has been tested for the case of stroke. Accordingly, we found that this was also true under the HIBD. In this study, overexpression of *TERT* promoted the proliferation of BMECs, formation of microvessels, and maintained BBB permeability in the brains of neonatal rats after HIBD. The underlining mechanism of this angiogenesis-promoting effect of TERT might be related with the Notch-1 signaling pathway. The neural regeneration capacity of the neonatal brain after injury is limited, which may be related to an unsuitable microenvironment and lack of proper nutritional support to the neural cells (Iwai et al., 2007). Angiogenesis not only improves microcirculation, but also promotes neurogenesis in the brain. As an important part of angiogenesis, endothelial cells can modulate neurogenesis after OGD *in vitro* (Plane et al., 2010). It has also been reported that endothelial cells can activate the self-renewal of neural stem cells by releasing soluble factors (Shen et al., 2004). Hence, post-injury angiogenesis is closely related to neurogenesis, both of which complement each other and play an important role in neural repair after HIBD.

##  Supplemental Information

10.7717/peerj.14220/supp-1 Supplemental Information 1 Raw data Immunofluorescence staining pictures, immunohistochemical staining pictures and uncropped gel photo, and the statistical analysis.

10.7717/peerj.14220/supp-2 Supplemental Information 2 ARRIVE 2.0 Checklist

## Additional Information and Declarations

### Data Availability

The following information was supplied regarding data availability: The raw data is available in the Supplemental Files.
