# Supplementary figures and images for: Telomerase reverse transcriptase promotes angiogenesis in neonatal rats after hypoxic-ischemic brain damage
Source: PeerJ. 2022 Oct 21;10:e14220. doi: 10.7717/peerj.14220 (PMC9590416; doi:10.7717/peerj.14220)

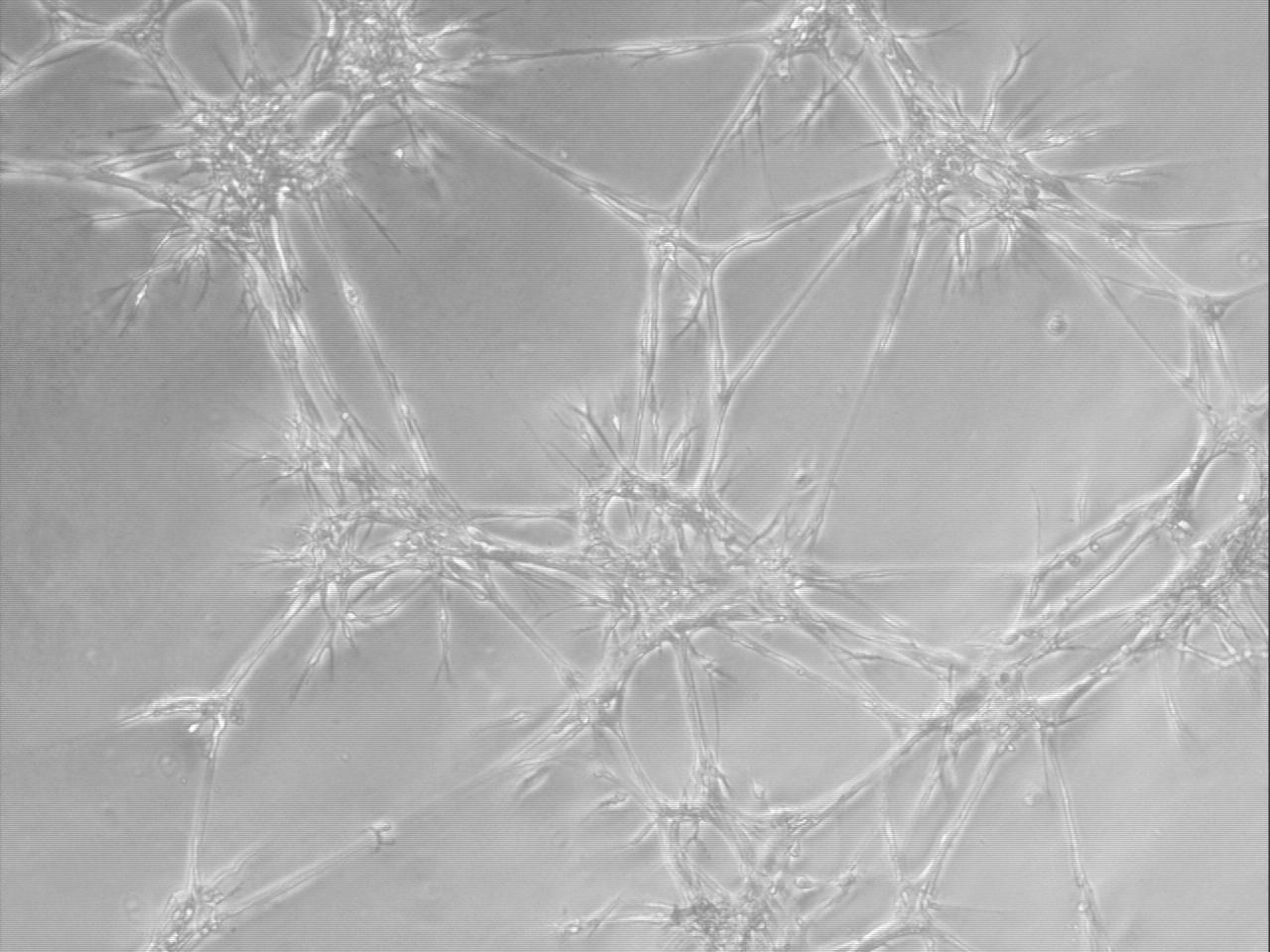

Supplement: Supplemental Information 1 — Immunofluorescence staining pictures, immunohistochemical staining pictures and uncropped gel photo, and the statistical analysis. [file peerj-10-14220-s001.zip › Supplementary_Material/figure2/control-light mirror.tif]

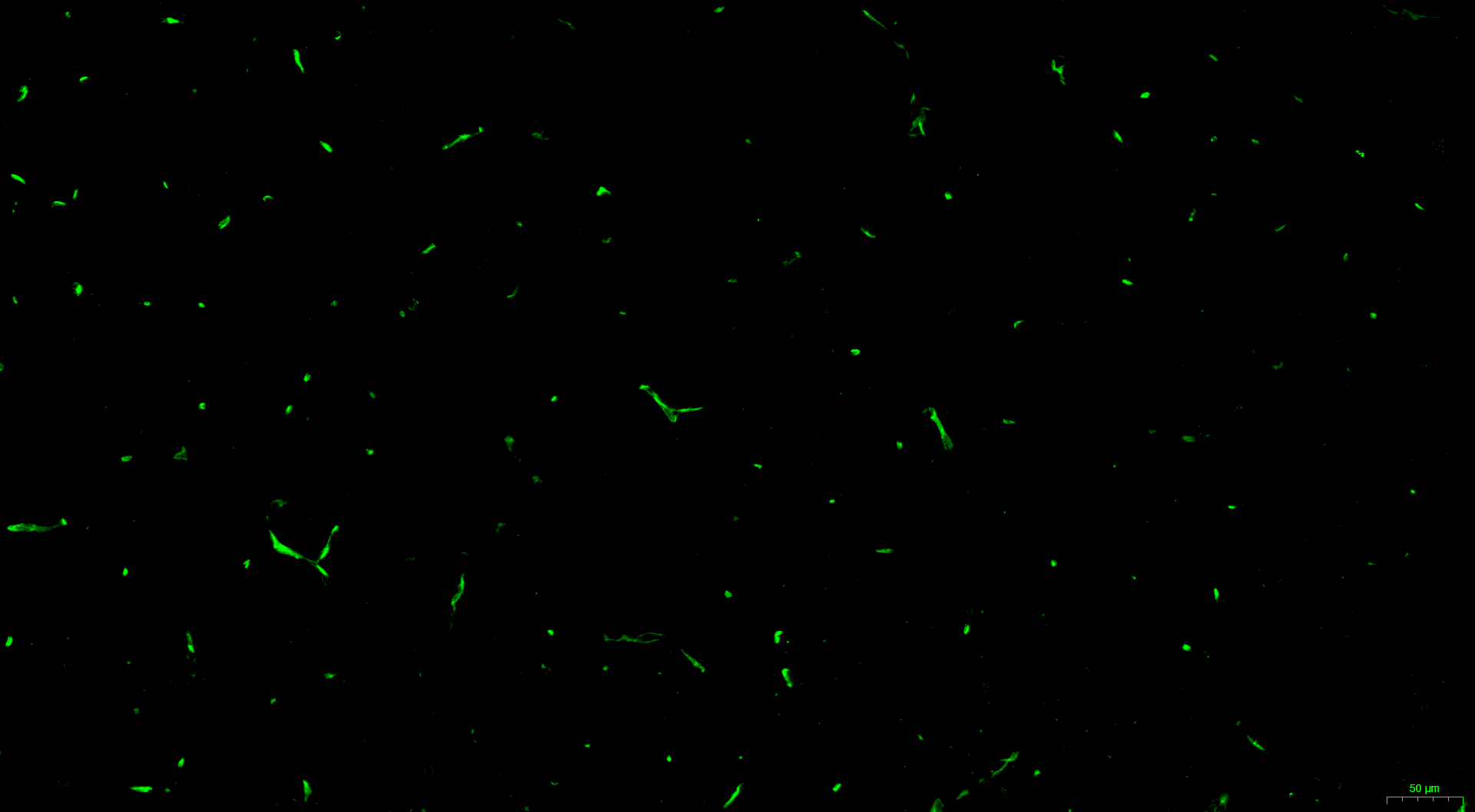

Supplement: Supplemental Information 1 — Immunofluorescence staining pictures, immunohistochemical staining pictures and uncropped gel photo, and the statistical analysis. [file peerj-10-14220-s001.zip › Supplementary_Material/figure2/No Load - CD34.tif]

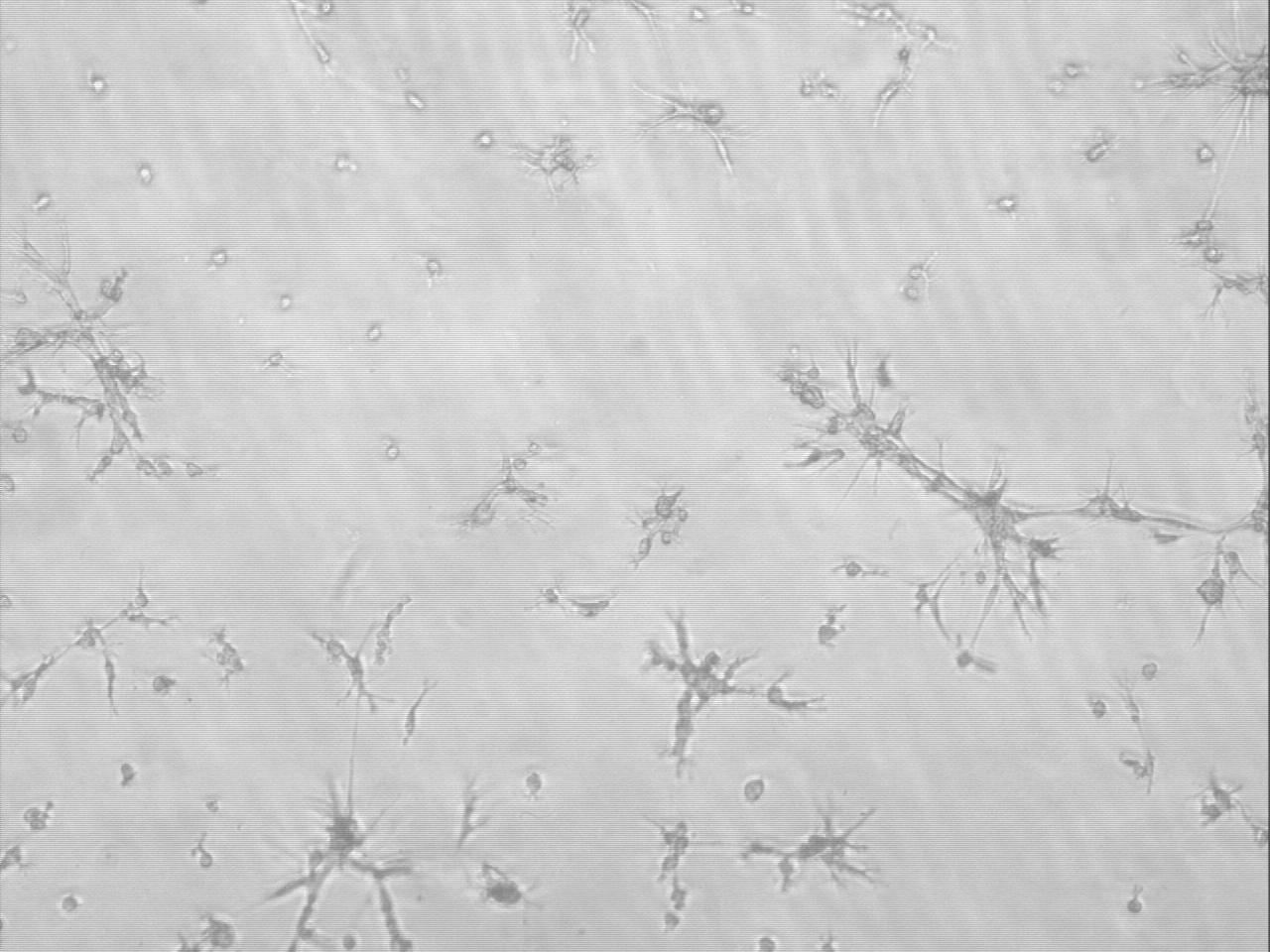

Supplement: Supplemental Information 1 — Immunofluorescence staining pictures, immunohistochemical staining pictures and uncropped gel photo, and the statistical analysis. [file peerj-10-14220-s001.zip › Supplementary_Material/figure2/No Load - Light Mirror.tif]

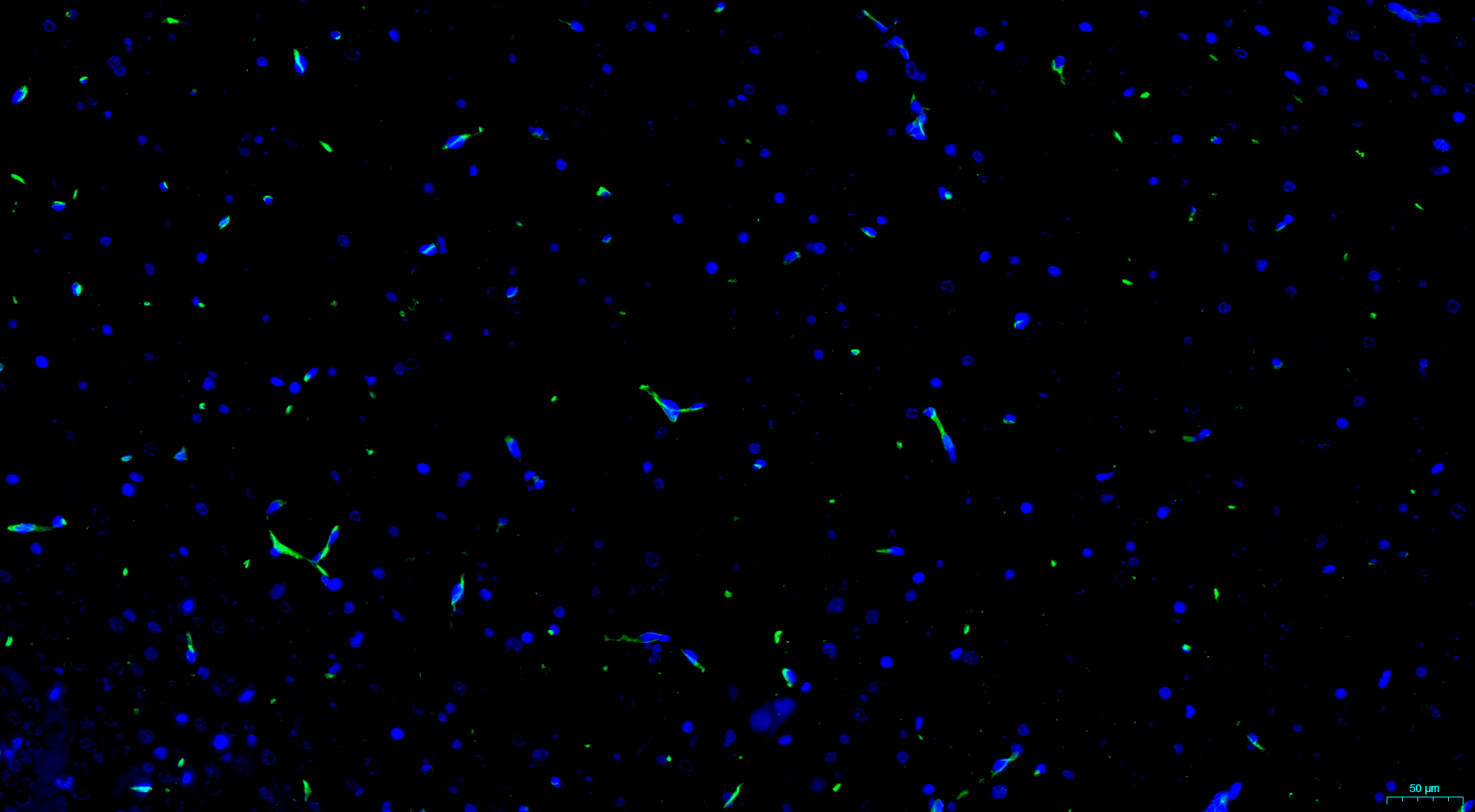

Supplement: Supplemental Information 1 — Immunofluorescence staining pictures, immunohistochemical staining pictures and uncropped gel photo, and the statistical analysis. [file peerj-10-14220-s001.zip › Supplementary_Material/figure2/No Load - MERGE.tif]

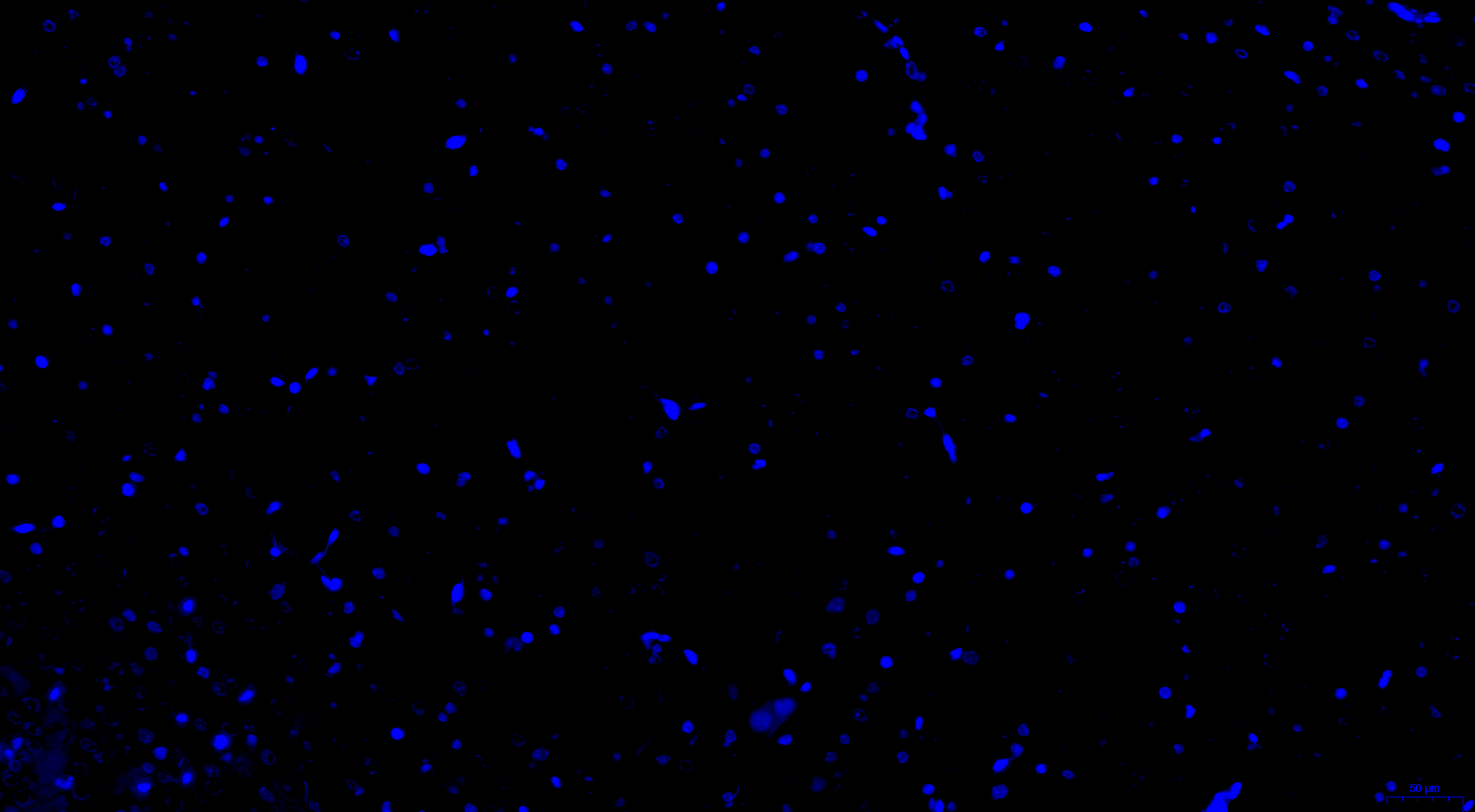

Supplement: Supplemental Information 1 — Immunofluorescence staining pictures, immunohistochemical staining pictures and uncropped gel photo, and the statistical analysis. [file peerj-10-14220-s001.zip › Supplementary_Material/figure2/No Load-DAPI.tif]

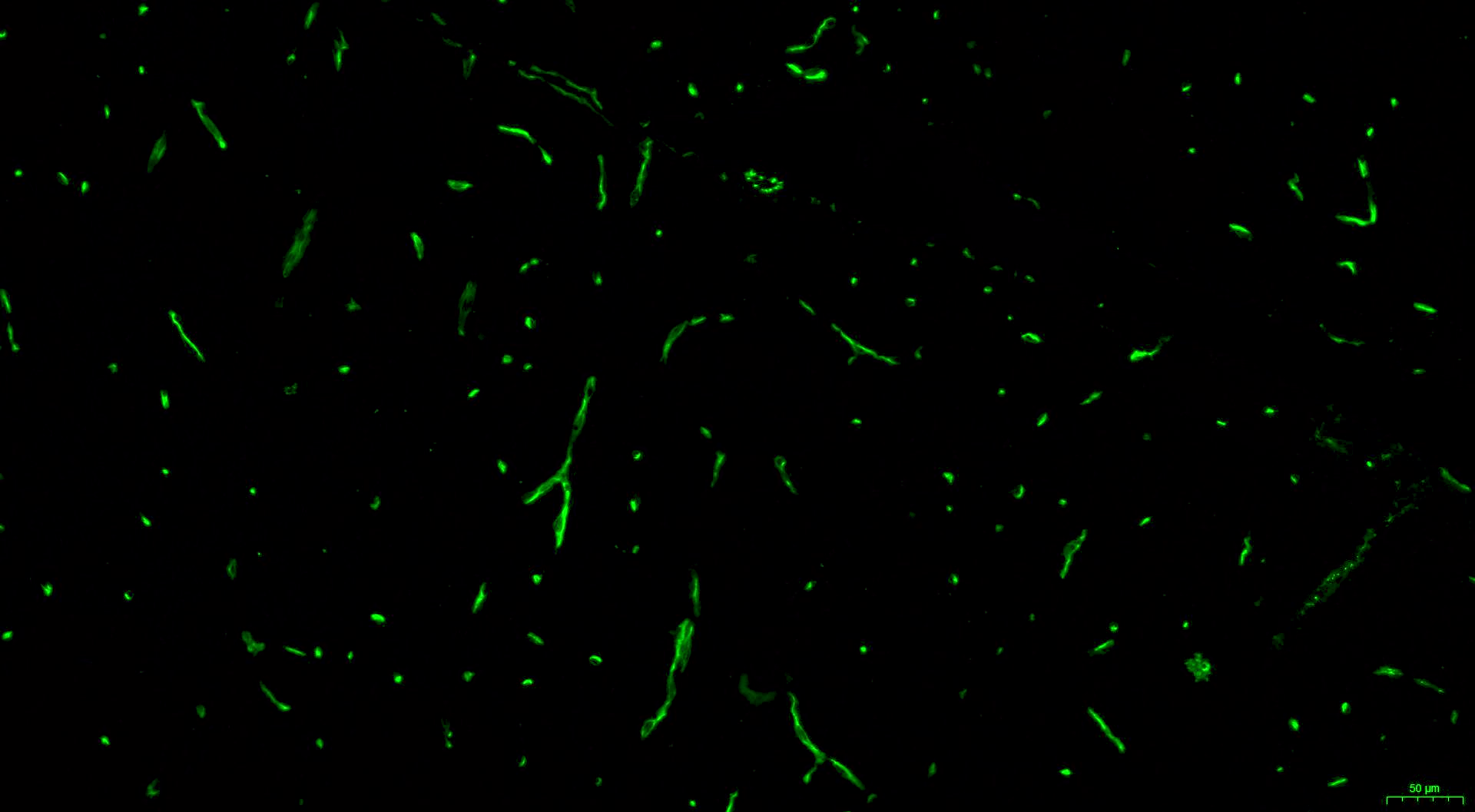

Supplement: Supplemental Information 1 — Immunofluorescence staining pictures, immunohistochemical staining pictures and uncropped gel photo, and the statistical analysis. [file peerj-10-14220-s001.zip › Supplementary_Material/figure2/SHAM-CD34.tif]

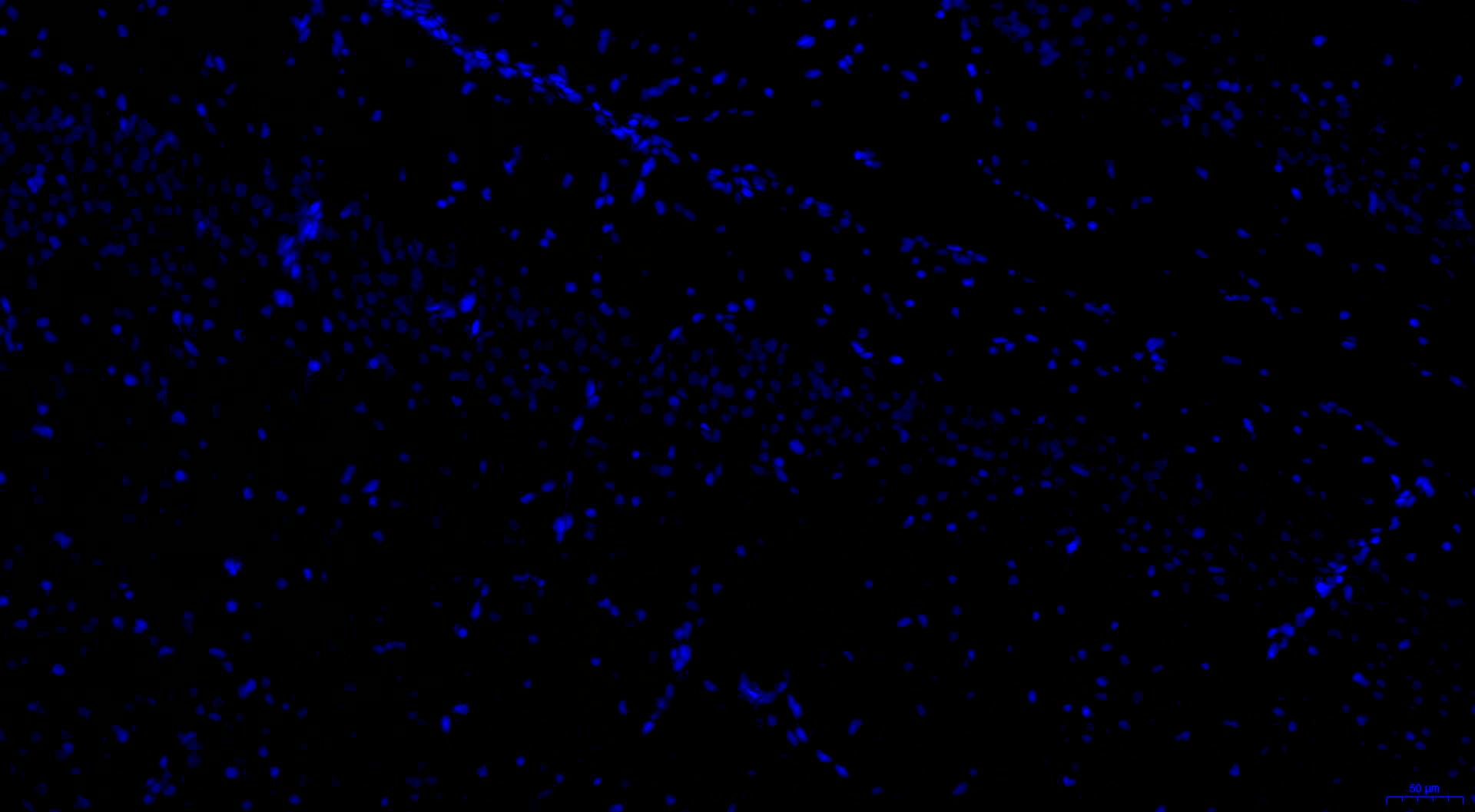

Supplement: Supplemental Information 1 — Immunofluorescence staining pictures, immunohistochemical staining pictures and uncropped gel photo, and the statistical analysis. [file peerj-10-14220-s001.zip › Supplementary_Material/figure2/SHAM-DAPI.tif]

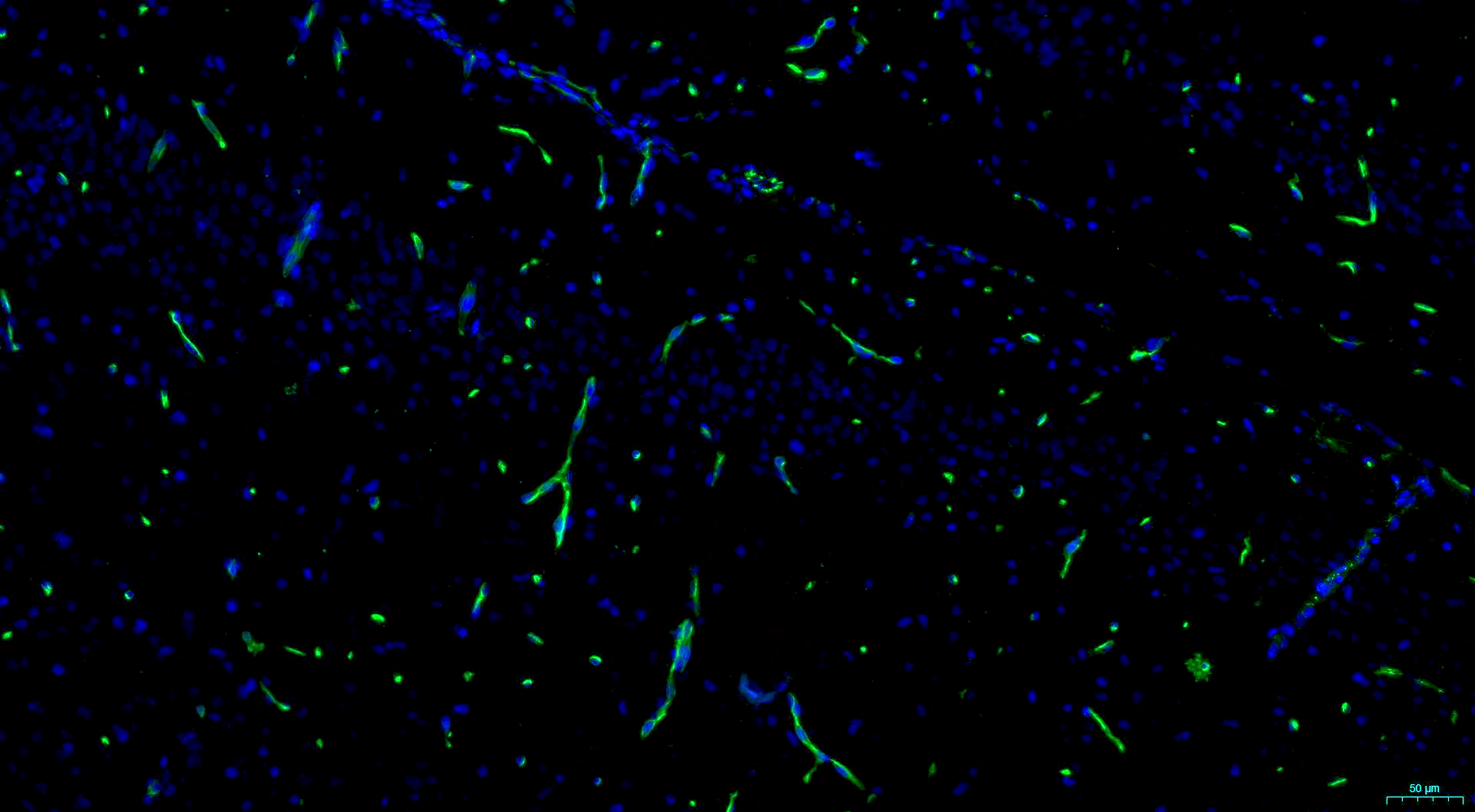

Supplement: Supplemental Information 1 — Immunofluorescence staining pictures, immunohistochemical staining pictures and uncropped gel photo, and the statistical analysis. [file peerj-10-14220-s001.zip › Supplementary_Material/figure2/SHAM-MERGE.tif]

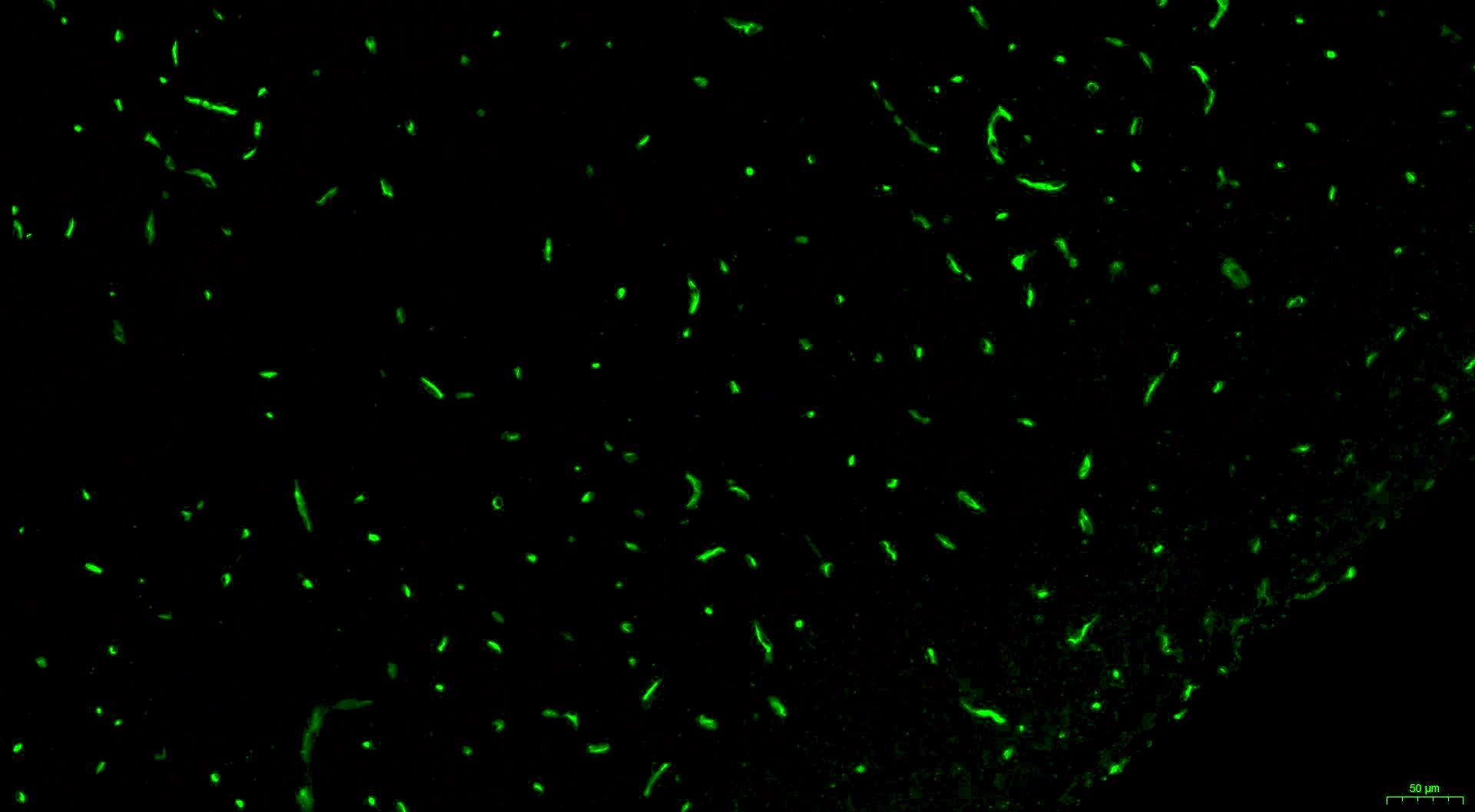

Supplement: Supplemental Information 1 — Immunofluorescence staining pictures, immunohistochemical staining pictures and uncropped gel photo, and the statistical analysis. [file peerj-10-14220-s001.zip › Supplementary_Material/figure2/TERT-CD34.tif]

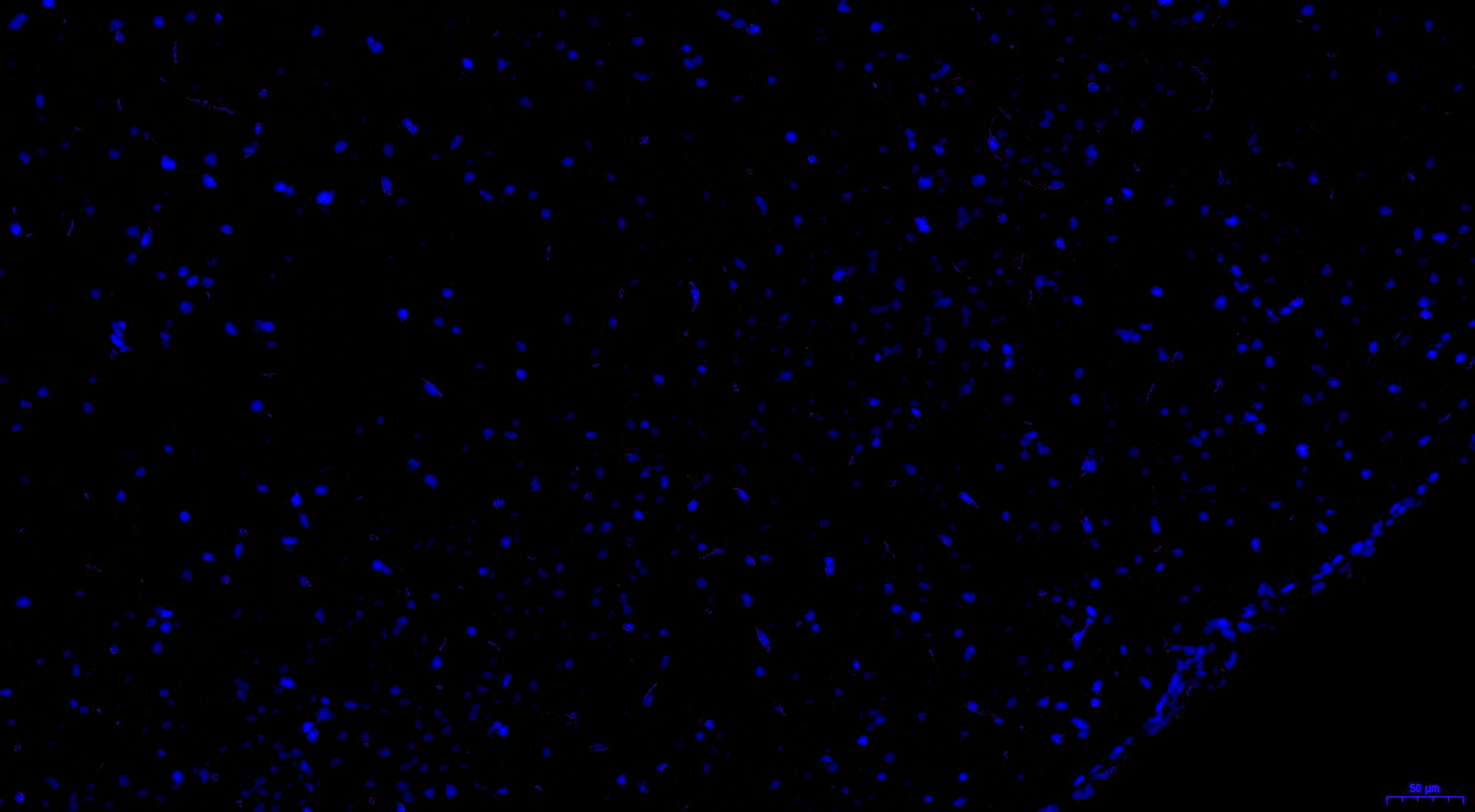

Supplement: Supplemental Information 1 — Immunofluorescence staining pictures, immunohistochemical staining pictures and uncropped gel photo, and the statistical analysis. [file peerj-10-14220-s001.zip › Supplementary_Material/figure2/TERT-DAPI.tif]

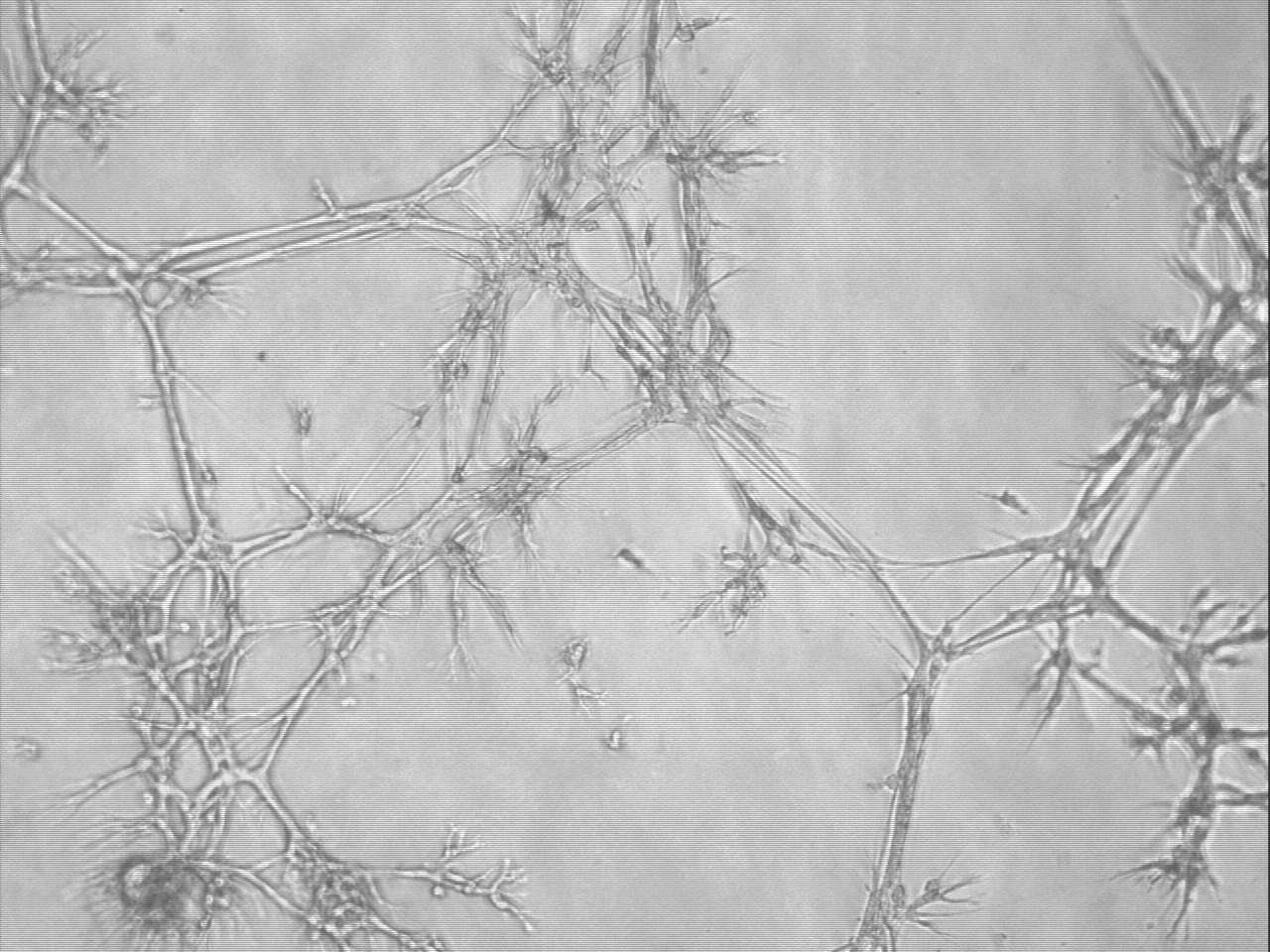

Supplement: Supplemental Information 1 — Immunofluorescence staining pictures, immunohistochemical staining pictures and uncropped gel photo, and the statistical analysis. [file peerj-10-14220-s001.zip › Supplementary_Material/figure2/TERT-Light Mirror.tif]

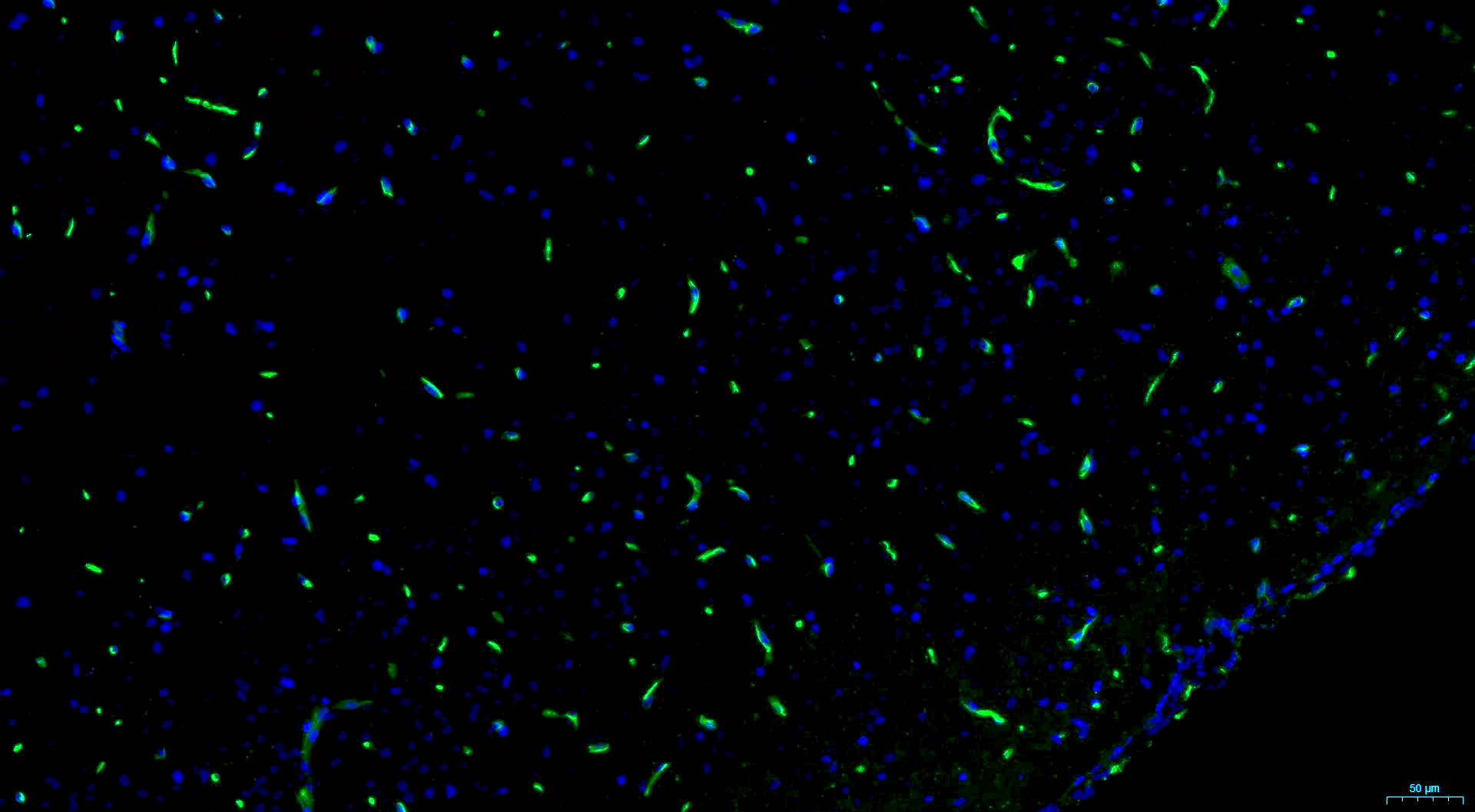

Supplement: Supplemental Information 1 — Immunofluorescence staining pictures, immunohistochemical staining pictures and uncropped gel photo, and the statistical analysis. [file peerj-10-14220-s001.zip › Supplementary_Material/figure2/TERT-merge.tif]

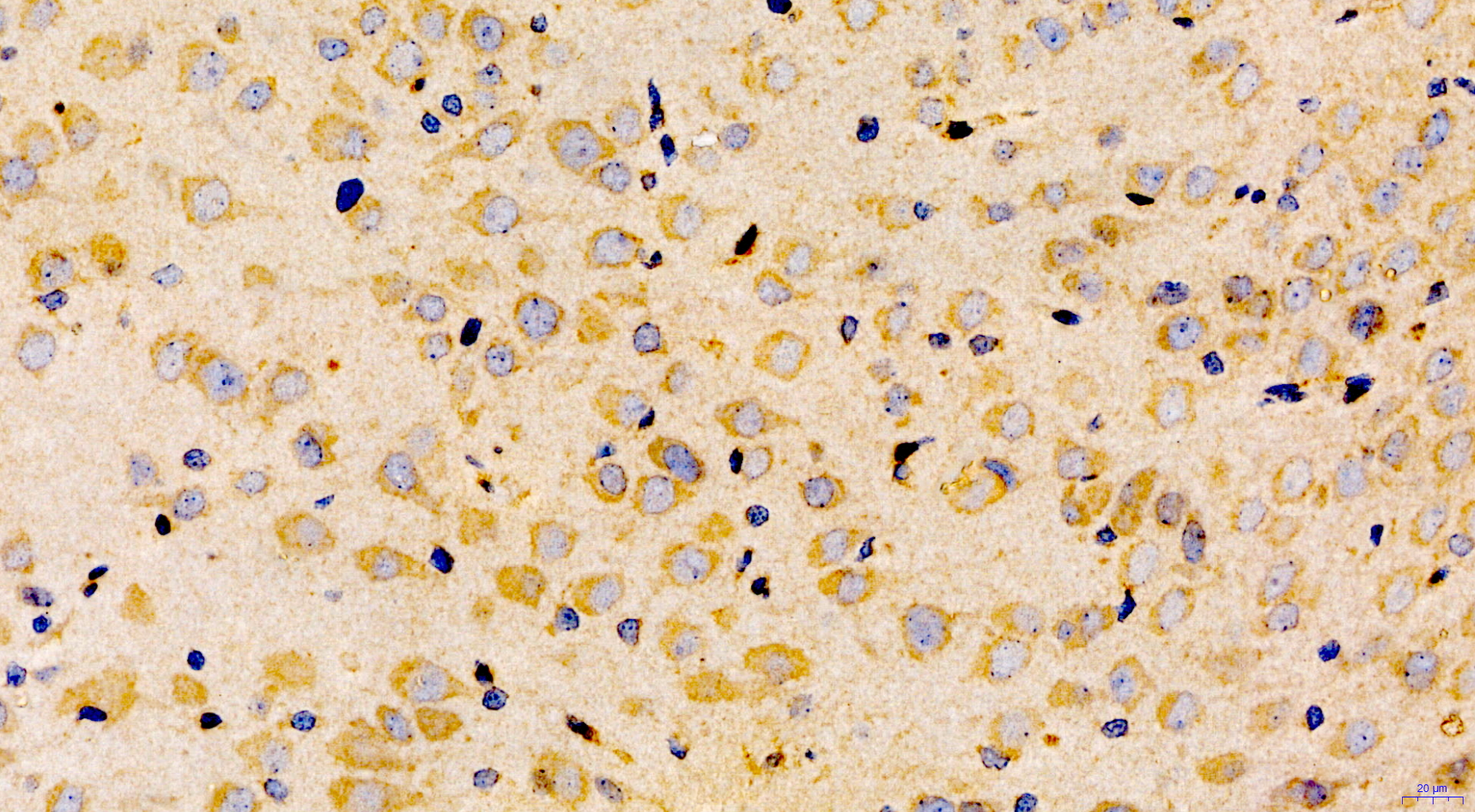

Supplement: Supplemental Information 1 — Immunofluorescence staining pictures, immunohistochemical staining pictures and uncropped gel photo, and the statistical analysis. [file peerj-10-14220-s001.zip › Supplementary_Material/figure3/Fake Surgery - 10_40.0x.tif]

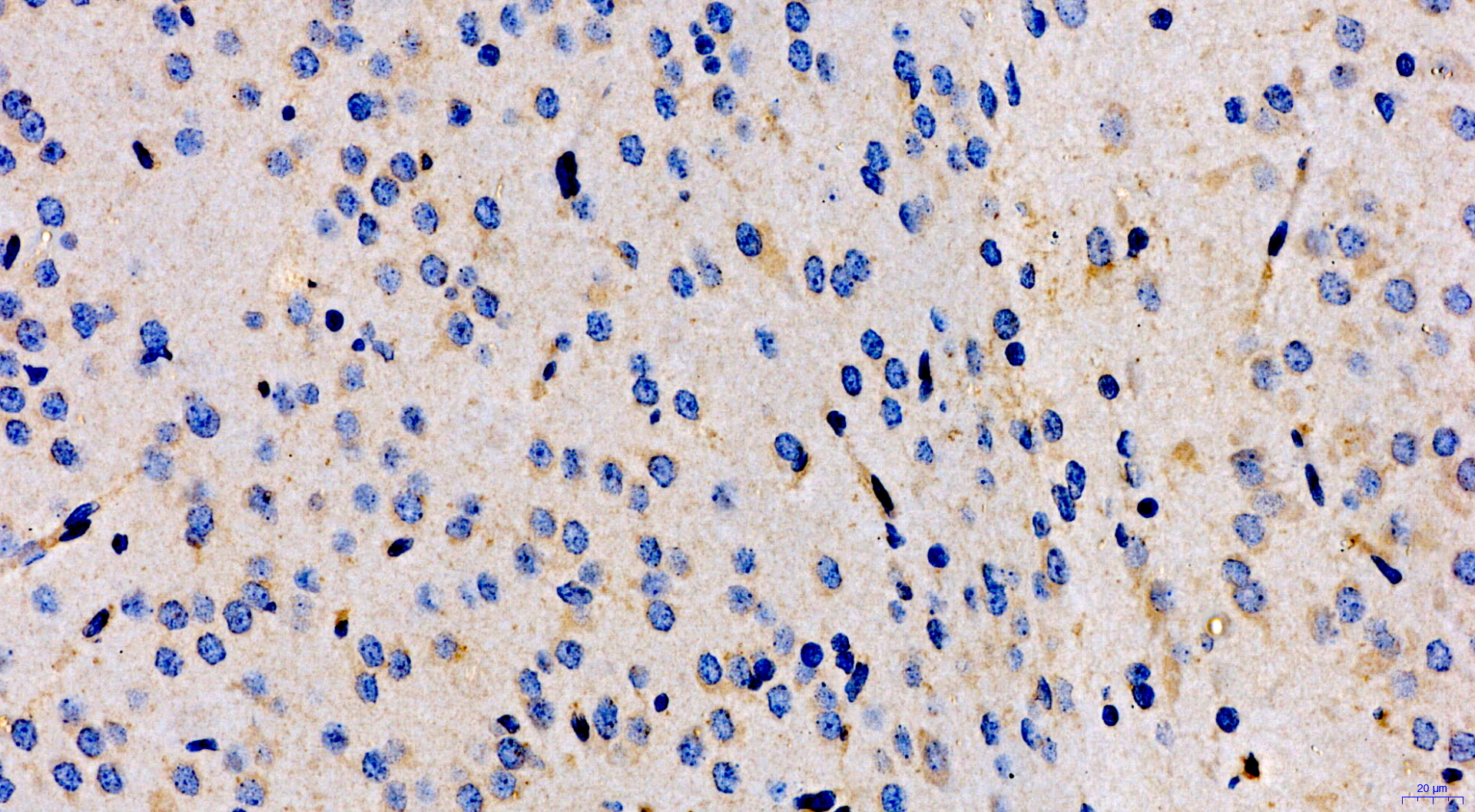

Supplement: Supplemental Information 1 — Immunofluorescence staining pictures, immunohistochemical staining pictures and uncropped gel photo, and the statistical analysis. [file peerj-10-14220-s001.zip › Supplementary_Material/figure3/No load 10_40.0x.jpg]

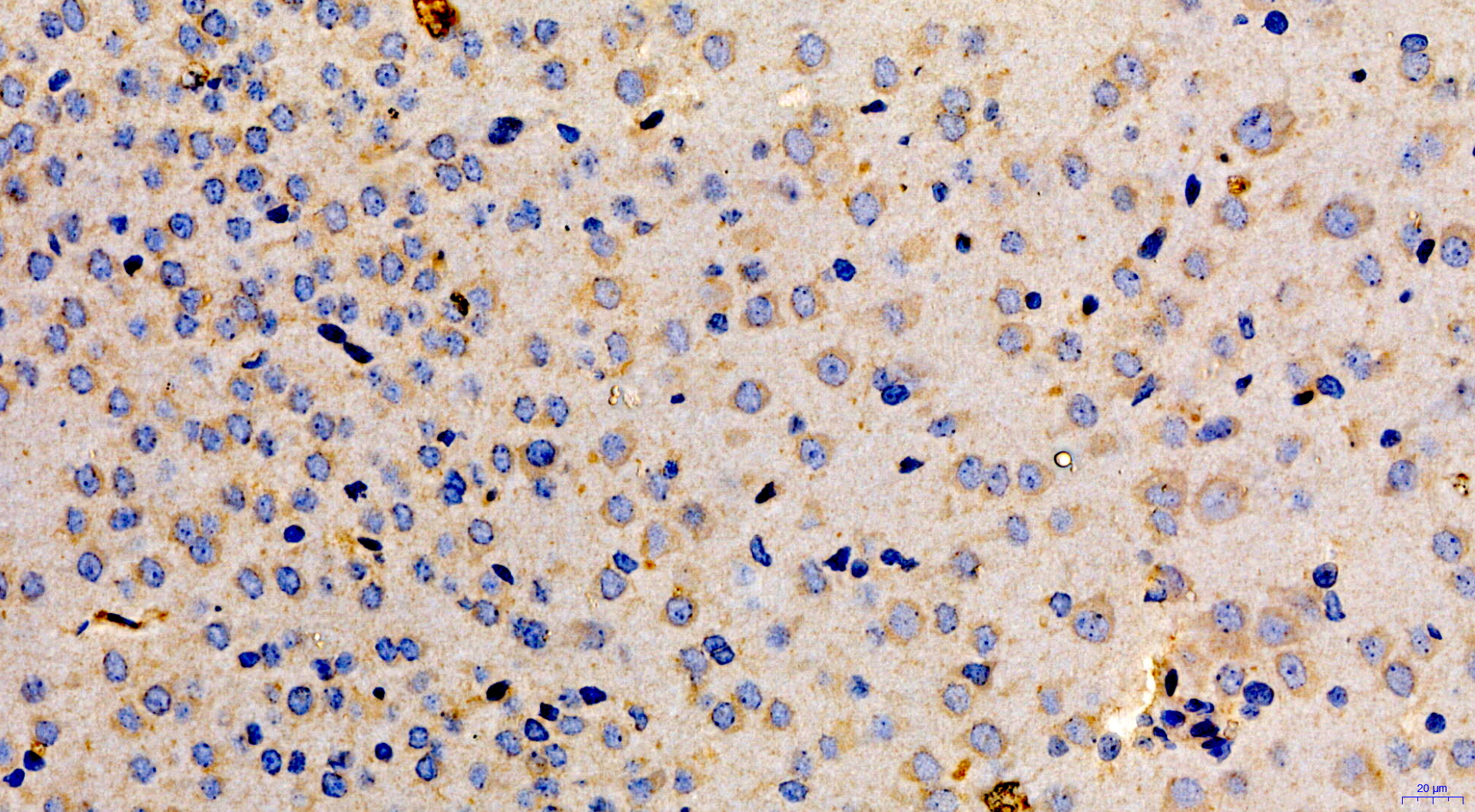

Supplement: Supplemental Information 1 — Immunofluorescence staining pictures, immunohistochemical staining pictures and uncropped gel photo, and the statistical analysis. [file peerj-10-14220-s001.zip › Supplementary_Material/figure3/TERT-7_40.0x.jpg]

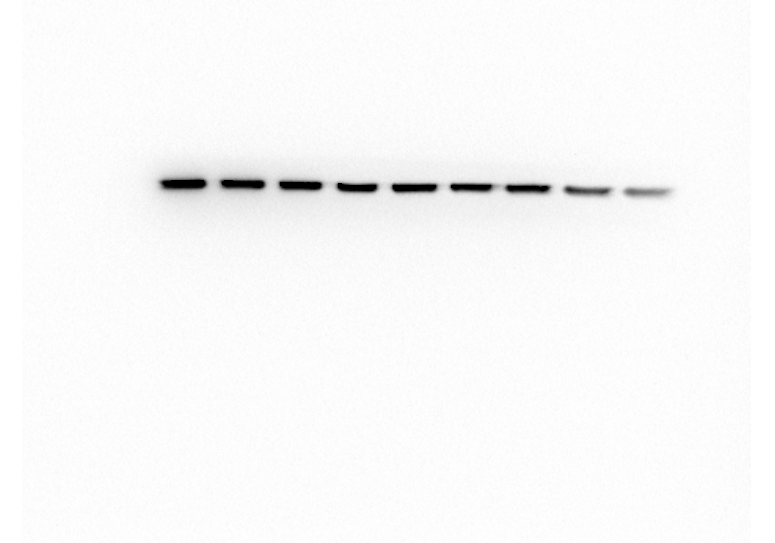

Supplement: Supplemental Information 1 — Immunofluorescence staining pictures, immunohistochemical staining pictures and uncropped gel photo, and the statistical analysis. [file peerj-10-14220-s001.zip › Supplementary_Material/figure4/Uncropped gel photo of B-actin.tif]

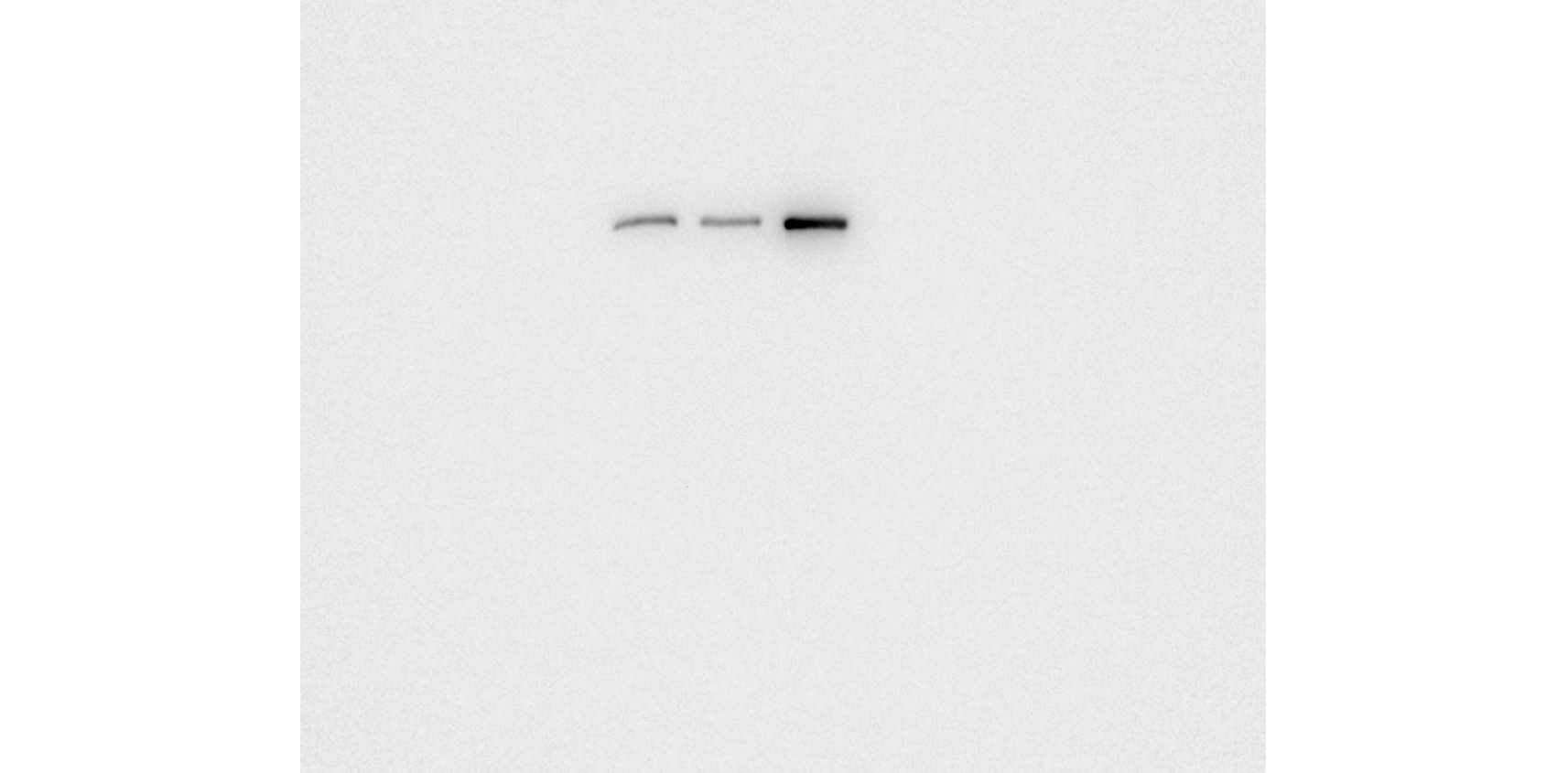

Supplement: Supplemental Information 1 — Immunofluorescence staining pictures, immunohistochemical staining pictures and uncropped gel photo, and the statistical analysis. [file peerj-10-14220-s001.zip › Supplementary_Material/figure4/Uncropped gel photo of Notch-1.tif]
